# Structural basis of DNA binding by YdaT, a functional equivalent of the CII repressor in the cryptic prophage CP-933P from *Escherichia coli* O157:H7

**DOI:** 10.1107/S2059798323001249

**Published:** 2023-02-27

**Authors:** Maruša Prolič-Kalinšek, Alexander N. Volkov, San Hadži, Jeroen Van Dyck, Indra Bervoets, Daniel Charlier, Remy Loris

**Affiliations:** a Structural Biology Brussels, Vrije Universiteit Brussel, Pleinlaan 2, 1050 Brussel, Belgium; b VIB–VUB Center for Structural Biology, Vlaams Instituut voor Biotechnologie, Pleinlaan 2, 1050 Brussel, Belgium; c Jean Jeener NMR Center, Vrije Universiteit Brussel, Pleinlaan 2, 1050 Brussel, Belgium; d Department of Physical Chemistry, Faculty of Chemistry and Chemical Technology, University of Ljubljana, Večna pot 113, 1000 Ljubljana, Slovenia; e Department of Chemistry, Universiteit Antwerpen, Groenenborgerlaan 171, 2020 Antwerpen, Belgium; f Research Group of Microbiology, Vrije Universiteit Brussel, Pleinlaan 2, 1050 Brussel, Belgium; Lund University, Sweden

**Keywords:** CII repressors, DNA binding, YdaT, transcription regulation, toxin–antitoxin

## Abstract

YdaT is a functional equivalent of the CII repressor in certain lambdoid phages and prophages. YdaT from the cryptic prophage CP-933P in the genome of *Escherichia coli* O157:H7 is functional as a DNA-binding protein and recognizes a 5′-TTGATTN_6_AATCAA-3′ inverted repeat. The DNA-binding domain is a helix–turn–helix (HTH)-containing POU domain and is followed by a long α-helix (α6) that forms an antiparallel four-helix bundle, creating a tetramer. The loop between helix α2 and the recognition helix α3 in the HTH motif is unusually long compared with typical HTH motifs, and is highly variable in sequence and length within the YdaT family. The POU domains have a large degree of freedom to move relative to the helix bundle in the free structure, but their orientation becomes fixed upon DNA binding.

## Abstract 2

YdaT, a functional equivalent of the bacteriophage λ CII repressor in certain lambdoid phages, is a member of the POU-domain family and recognizes a 5′-TTGATTN_6_AATCAA-3′ inverted repeat.

## 1. Introduction

Since the early days of molecular biology, *Escherichia coli* bacteriophage λ has served as a model organism and as a tool in molecular genetics. The ‘lysis versus lysogeny’ decision of the phage has been studied in great detail and serves as a paradigm for regulatory gene circuits (Wegrzyn & Wegrzyn, 2005 ▸). The circuit, which depends on three transcription factors (CI, CII and Cro), results in bistability. The interplay between CI and Cro determines whether a λ prophage remains inserted in the host chromosome or start a lytic cycle (Ptashne *et al.*, 1980 ▸; Johnson *et al.*, 1981 ▸). CII, on the other hand, is essential in order to enter the lysogenic path immediately after infection (Chung & Echols, 1977 ▸). CII is expressed after infection of *E. coli* with λ, with its expression level depending on both cellular and environmental factors that affect the half-life of this rather unstable protein (Kobiler *et al.*, 2002 ▸). When its expression level crosses a certain threshold, it directs the lysis/lysogeny decision towards lysogeny. CII activates three promotors on the phage genome: *P_I_*, *P_RE_* and *P_AQ_*. From *P_AQ_* an antisense RNA is transcribed that prevents production of the Q protein to reduce lytic activity until the repressor CI is sufficiently expressed (Ho & Rosenberg, 1985 ▸). CI is transcribed from *P_RE_*, resulting in the shutdown of all lytic genes. Finally, from the *P_I_* promotor, the Int protein is produced that integrates the phage genome into the *E. coli* chromosome (Court *et al.*, 2007 ▸). Once lysogeny has been established, CII is no longer required and remains turned off.

Lambdoid phages are a family of bacteriophages related to coliphage λ, with which they can form viable recombinants. Most lambdoid phages grow on *E. coli*, but a few such as P22 come from *Salmonella typhimurium*. They are highly polymorphic in DNA sequence and biological specificity, with differences observed in receptor specificity, integration and the mechanism of packaging (Campbell, 1994 ▸). Different *E. coli* strains typically contain several cryptic lambdoid phages in their genomes. For example, *E. coli* O157:H7 Sakai contains 18 such prophage genome elements that together constitute about 16% of its total genomic DNA content (Brüssow & Kutter, 2004 ▸).

Among the different defective prophages (that are missing one or more crucial genes to allow initiation of a lytic cycle) present in the genome of *E. coli* O157:H7 is CP-933P (Perna *et al.*, 2001 ▸). It contains a three-component version of the *parDE* type of toxin–antitoxin module termed *paaR2–paaA2–parE2* next to the *ydaST* gene pair (Hallez *et al.*, 2010 ▸). The *ydaS* and *ydaT* genes were initially suspected (from an analysis using the *RASTA-Bacteria* algorithm) to constitute a toxin–antitoxin operon in *E. coli* O157:H7, with YdaT supposedly being the toxin (Sevin & Barloy-Hubler, 2007 ▸). Experimental evidence nevertheless failed to prove this hypothesis (Christensen-Dalsgaard *et al.*, 2010 ▸). Indeed, their genetic context pinpoints *ydaS* and *ydaT* as equivalents of *cro* and *cII*, respectively (Casjens, 2003 ▸; Jobling, 2018 ▸; Jurėnas *et al.*, 2021 ▸). Homologs are found in a number of *rac* prophages, where their expression is repressed by RacR (Krishnamurthi *et al.*, 2017 ▸). Rac phages are a group of defective prophages that are found in various *E. coli* strains, with Rac itself being the first defective prophage to be discovered in *E. coli* K-12 (Kaiser & Murray, 1979 ▸). The CI repressor is here typically referred to as ‘RacR’.

**Figure 1:**
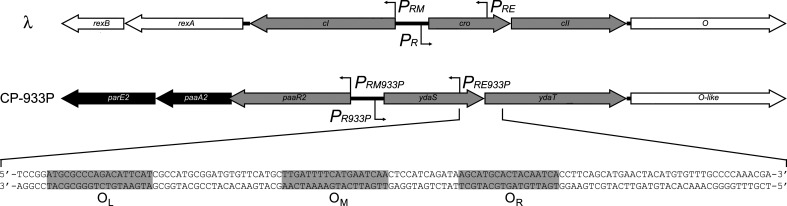
Comparison of the immunity regions of bacteriophage λ and *E. coli* O157:H7 prophage CP-933P. Genes are shown as arrows, with the direction of the arrow indicating the direction of transcription. Promoters of λ and their CP-933P equivalents are indicated. The three repressors are coloured grey, while other neighbouring phage genes are white. The toxin–antitoxin genes *parE2* and *paaA2* in CP-933P are coloured black and replace *rexB* and *rexA*, respectively, in λ. The border sequence of the interface between the *ydaS* and *ydaT* genes is highlighted and the three YdaT binding sites O_L_, O_M_ and O_R_ are boxed in grey and labelled. The O_M_ sequence also contains an alternative transcription start for the *paaR2–paaA2–parE2* operon that is controlled via YdaT (Jurėnas *et al.*, 2021 ▸).

A schematic comparison between the immunity regions of bacteriophage λ and CP-933P is given in Fig. 1 ▸. Similar to λ CII, which binds the *P_RE_* promotor located between the λ *cro* and *cII* genes and activates transcription from *P_RE_*, YdaT is predicted to bind at the interface between the *ydaS* and *ydaT* genes. This region contains the *P_RE993P_* promotor, the CP-933P equivalent of λ *P_RE_*. Although basal transcriptional activity from *P_RE993P_* is very weak, overexpression of YdaT increases this activity significantly (Jurėnas *et al.*, 2021 ▸).

Although both YdaS and YdaT are predicted to contain a helix–turn–helix motif and serve a similar function as the λ Cro and CII proteins, respectively (Jurėnas *et al.*, 2021 ▸), the YdaS and YdaT proteins show no detectable sequence similarity to λ Cro or CII. YdaT proteins constitute a family of transcription factors that currently remain uncharacterized in terms of structure and DNA-binding activity. In order to better understand how YdaT functions at the molecular level, we determined the crystal structure of CP-933P YdaT and identified its exact binding site as three regions, O_L_, O_M_ and O_R_, between the *ydaS* and *ydaT* genes. Of these, O_M_ covers the alternative transcription start for the *paaR2–paaA2–parE2* operon as identified by Jurėnas *et al.* (2021 ▸). We furthermore created and validated a model for the interaction between YdaT and its operator. Together, our results paint a consistent picture of the functioning of YdaT repressors.

## 2. Materials and methods

### 2.1. Cloning, expression and purification

Plasmid pET-28b containing the open reading frame for YdaT from *E. coli* O157:H7 (UniProt ID A0A6M7H0F8) with an N-terminal His tag (GSSHHHHHHSSG) was transformed into competent *E. coli* BL21 (DE3) cells (Table 1 ▸). Transformed cells were plated on agar plates supplemented with kanamycin (25 µg ml^−1^) and incubated at 37°C overnight. LB medium supplemented with kanamycin (25 µg ml^−1^) was inoculated with one colony and left to incubate overnight at 37°C while shaking at 130 rev min^−1^. 5 ml of the overnight culture was added to 500 ml LB medium (supplemented with 25 µg ml^−1^ kanamycin) in 2 l flasks and incubated at 37°C with shaking at 130 rev min^−1^. When the OD_600_ reached 0.6–0.8, protein expression was induced with 0.5 m*M* isopropyl β-d-1-thiogalactopyranoside (IPTG). Upon induction, the cultures were incubated at 37°C for 4 h, centrifuged at 5000 rev min^−1^ for 15 min, resuspended in lysis buffer [20 m*M* Tris–HCl, 500 m*M* NaCl, 20 m*M* MgCl_2_ pH 8.0, 0.1 mg ml^−1^ 4-(2-aminoethyl)benzenesulfonyl fluoride hydrochloride (AEBSF)] and stored at −80°C. To purify the protein, the frozen cells were thawed and DNase I was added (50 µg ml^−1^). The cells were lysed by sonication (5 min, 5 s on and 5 s off, 70% amplitude) and the lysate was centrifuged at 18 000 rev min^−1^ for 45 min. The supernatant was filtered (0.45 µm HAWP filter) and loaded onto a pre-packed HisTrap HP Ni^2+^-Sepharose column (GE Healthcare) pre-equilibrated in 20 m*M* Tris–HCl, 500 m*M* NaCl, 5 m*M* imidazole pH 8.0. The column was then washed with the same buffer until baseline stabilization. A linear gradient (0.0–1.0 *M* imidazole in 20 column volumes) elution was applied in 20 m*M* Tris–HCl, 500 m*M* NaCl, 1 *M* imidazole pH 8.0. Fractions containing the protein were concentrated and loaded onto a Superdex 200 16/90 size-exclusion chromatography (SEC) column (GE Healthcare) pre-equilibrated in 20 m*M* Tris–HCl, 500 m*M* NaCl pH 8.0. The purity of the protein was assessed by SDS–PAGE.

A truncated variant of YdaT lacking the 45 C-terminal residues (YdaT^1–96^) was created by replacing Ser97 with a stop codon (TAA) via PCR amplification of the whole plasmid using primers 1 and 2 (Table 1 ▸, Supplementary Table S1) and Q5 High-Fidelity 2× Master Mix (NEB). The unmodified plasmid was degraded by incubation with DpnI for 1 h at 37°C. The mutations were confirmed by sequencing and CaCl_2_-competent *E. coli* BL21 Star (DE3) cells were transformed with the mutated plasmids. Both proteins were expressed and purified as described for wild-type YdaT except for the use of a Superdex 75 16/60 SEC column (GE Healthcare) for the SEC purification step.

### 2.2. Concentration determination

The concentrations of the samples were determined by measuring the UV absorbance at 280 nm for the proteins and at 260 nm for the oligonucleotides. Extinction coefficients for the proteins were calculated using *ProtParam* (Gasteiger *et al.*, 2005 ▸; https://web.expasy.org/protparam/) and are 19 940 *M* ^−1^ cm^−1^ for YdaT and 15 470 *M* ^−1^ cm^−1^ for YdaT^1–96^. For DNA, extinction coefficients were calculated according to Tataurov *et al.* (2008 ▸).

### 2.3. X-ray crystallography

Crystals of YdaT were produced by a sitting-drop method using three-well Intelli-Plates and a Mosquito robot. 100 nl YdaT protein (13.23 mg ml^−1^ in 20 m*M* Tris, 200 m*M* NaCl pH 7.5) was mixed with 100 nl reservoir solution and equilibrated against 70 µl reservoir solution from various commercial crystallization kits at 19°C. Crystals grew in 0.1 *M* bis-Tris pH 5.5, 2.0 *M* ammonium sulfate. The crystals were harvested, dipped in cryoprotectant solution [0.1 *M* bis-Tris pH 5.5, 2.0 *M* ammonium sulfate, 22.5%(*v*/*v*) glycerol] and vitrified in liquid nitrogen.

Diffraction data were collected on beamline PROXIMA-1 at the SOLEIL synchrotron, Gif-sur-Yvette, France and were recorded on an EIGER X 9M photon-counting area detector. The data were indexed, scaled and merged using *XDS* (Kabsch, 2010 ▸). The data were further corrected for anisotropy with the *STARANISO* server (Tickle *et al.*, 2018 ▸) using the ‘unmerged data’ protocol. The data were limited to 240° total crystal rotation due to radiation damage.

The structure of YdaT was determined by molecular replacement using *Phaser* as implemented in the *CCP*4 package (McCoy *et al.*, 2007 ▸). The crystal structure of PDB entry 3c4r (sequence identity of 84%; annotated as an uncharacterized protein from a cryptic prophage in *E. coli* O6:H1; New York SGX Research Center for Structural Genomics, unpublished work) was used as the search model. The initial solution was refined with *phenix.refine* using a maximum-likelihood target against intensities (Afonine *et al.*, 2012 ▸) and was rebuilt with *Coot* (Emsley *et al.*, 2010 ▸). NCS restraints were applied throughout the refinement procedure and the final cycles involved refinement of TLS parameters. Data-collection and refinement statistics are summarized in Table 2 ▸ and full details are given in Supplementary Tables S2 and S3.

### 2.4. Circular-dichroism spectroscopy

Measurements were performed on a Jasco J-750 spectrophotometer (Jasco, Japan). Spectra were collected from 200 to 250 nm every 1 nm, with a bandwith of 1 nm, a scan rate of 50 nm min^−1^ and five acquisitions, in a quartz cuvette (1 mm optical path length). The protein concentration for all samples was 0.2 mg ml^−1^. Data were normalized to obtain the mean residue ellipticity ([θ] in deg cm^2^ dmol^−1^) using the formula

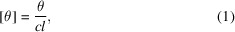

where θ is the ellipticity (raw data), *c* is the molar concentration and *l* is the optical path length. The buffer spectrum was subtracted from the sample spectra. Protein solutions were prepared in 20 m*M* Tris–HCl, 500 m*M* NaCl pH 8.0 buffer.

### 2.5. Size-exclusion chromatography and multi-angle light scattering (SEC-MALS)

YdaT was dialysed overnight against 20 m*M* Tris–HCl, 200 m*M* NaCl pH 7.5. The buffer was filtered through a 0.1 µm filter (Sartorius) three times before measurements. Protein samples were centrifuged at 17 000*g* for 10 min prior to injection. 20 µl of protein sample at a concentration of 1 or 10 mg ml^−1^ was injected into a Shodex KW402.5-4F column (Showa Denko K. K.) that had been pre-equilibrated with dialysis buffer on an Alliance e2695 XE HPLC System (Waters) connected to a TREOS II light-scattering detector (Wyatt Technology) and a Shodex RI-501 refractive-index detector (Showa Denko K. K.). The flow rate was set to 0.2 ml min^−1^. Data processing and molecular-weight calculations were performed using the *ASTRA V* software (Wyatt Technology). Bovine serum albumin at a concentration of 1 mg ml^−1^ was used as a standard for calibration.

### 2.6. Native mass spectrometry

YdaT was buffer-exchanged into 200 m*M* ammonium acetate pH 8 using Amicon Ultra 0.5 ml centrifugal filters (Merck Millipore) with a molecular-weight cutoff of 3 kDa. The concentrations of protein and oligonucleotide alone were 2.5 µ*M* (tetramer concentration) and 5 µ*M* (DNA duplex), respectively. The complexes between YdaT and oligonucleotide were prepared at different YdaT tetramer:DNA molar ratios (0.25:1, 0.25:1.5, 0.5:1, 0.75:1, 1:1, 1.25:1 and 1.5:1), keeping the protein concentration fixed at 2.5 µ*M* YdaT tetramer. Native mass spectrometry was performed on a Synapt G2 mass spectrometer (Waters). The samples were introduced into the gas phase through nano-electrospray ionization with in-house-prepared gold-coated borosilicate glass capillaries. The settings were optimized for the analysis of larger structures as natively as possible. The critical voltages and pressures used were a sampling cone voltage of 50 V and a trap collision energy of 10 V, with pressures throughout the instrument of 6.18 and 2.42 × 10^−2^ mbar for the source and trap collision cell regions, respectively. Analysis of the acquired spectra was performed using *MassLynx* version 4.1 (Waters). Native MS spectra were smoothed (to an extent depending on the size of the complexes) and additionally centred to calculate the molecular weights to determine precise stoichiometries.

### 2.7. Isothermal titration calorimetry

Oligonucleotides were purchased from Sigma and were HPLC-purified. Double-stranded DNA fragments were prepared by mixing single-stranded oligonucleotides corresponding to the upper and lower strands of the operator DNA in a 1:1 molar ratio followed by incubation at 95°C for 5 min in a water bath. They were subsequently left to cool to room temperature. The annealing was checked by native PAGE. Proteins and oligonucleotides were dialysed against 10 m*M* NaH_2_PO_4_, 10 m*M* Na_2_HPO_4_, 100 m*M* NaCl, 50 m*M* glutamic acid, 50 m*M* arginine pH 7.5 with two buffer changes followed by overnight dialysis. Prior to measurements, the samples were spun down at 13 300 rev min^−1^ for 10 min and degassed on a degassing station (TA Instruments) for 15 min.

The titrations were carried out at 25°C. The concentrations of the oligonucleotides in the sample cell were 5 µ*M*. The concentration of YdaT in the syringe ranged between 25 and 140 µ*M* calculated for YdaT as a tetramer. When O_M_ was titrated with YdaT, the concentration in the cell was 5 µ*M* and that in the syringe was 100 µ*M*. The measurements were performed on a VP-ITC microcalorimeter (MicroCal) or a MicroCal PEAQ-ITC microcalorimeter (Malvern Panalytical). The integration of thermograms was performed with *NITPIC* (Scheuermann & Brautigam, 2015 ▸).

For the binding of YdaT to O_M_, we assumed that YdaT contains two independent non-equivalent binding sites for O_M_. The corresponding binding reaction can be represented as

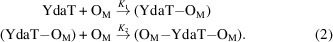




For the titrations of YdaT against O_LM_ and O_MR_ an additional binding step was included in the mechanism, since YdaT can also bind to the second site on the DNA,

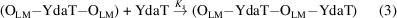

and 

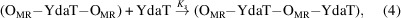




The values of the equilibrium constants *K*_1_, *K*_2_ and *K*_3_ and the corresponding enthalpies of reaction were obtained by fitting the appropriate model equation to the ITC data similarly to as described previously (Vandervelde *et al.*, 2017 ▸). A system of mass-balance equations was derived given the above reaction schemes. Given the total concentrations of reactants (YdaT and oligonucleotides) and the assumed values of the constants *K_i_*, we calculate the equilibrium concentrations of all molecular species using a root-solving routine. By calculating the composition of the experimental system at each point during titration we then calculate the model-based value of the enthalpy as

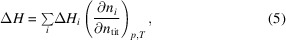

where Δ*H_i_* is the enthalpy of formation of the complex *i*, (∂*n_i_*/∂*n*_tit_)_*p*,*T*_ is the corresponding partial derivative at the given pressure *p* and temperature *T* in which *n_i_* is the amount of complex *i* and *n*_tit_ is the amount of added titrant (YdaT or oligonucleotide, depending on the titration). The parameters *K_i_* and *H_i_* were adjusted using the Nelder–Mead optimization algorithm to produce the best match between the model-calculated and experimental values of Δ*H*. The interactions of YdaT with O_M_ in both direct (protein to DNA) and reverse titrations were fitted simultaneously (global fit), while for interactions of YdaT with O_LM_ or O_MR_ only direct titrations were used for fitting.

### 2.8. Electrophoretic mobility shift assay (EMSA)

For radioactive EMSA, a 206 bp segment of DNA was used that corresponds to the end of the *ydaS* gene and the start of the *ydaT* gene. The DNA was generated by PCR using primers 5 and 6 (Supplementary Table S1) and genomic DNA of *E. coli* O157:H7 strain EDL933 as a template. One of the primers was labelled with γ-^32^-P-ATP using T4 polynucleotide kinase. The PCR fragment was purified from polyacrylamide gel and concentrated by ethanol precipitation. In the same way, a non­specific DNA was generated from genomic DNA of *Cupriavidus metallidurans* NA4 containing the promotor region of *prsQ2*. Protein and DNA were mixed in 20 m*M* Tris, 200 m*M* NaCl pH 7.5 in a total volume of 20 µl at different protein concentrations and left to incubate for 30 min at room temperature. The protein concentrations (tetramer equivalents) were in the range 2–30 µ*M*. DNA with 7500 cpm was used in each reaction. The samples were then loaded onto a 6% non­­denaturing polyacrylamide gel using 2 µl loading dye (25% Ficoll, 0.1% xylene cyanol, 0.1% bromophenol blue).

### 2.9. DNase I footprinting

For DNase I footprinting, a 236 bp segment of DNA corresponding to the end of the *ydaS* gene and the start of the *ydaT* gene was generated by PCR using primers 5 and 7 (Supplementary Table S1) as described above with genomic DNA of *E. coli* O157:H7 strain EDL933 as a template. One of the primers was labelled with γ-^32^P-ATP using T4 polynucleotide kinase. The PCR fragment was purified from polyacrylamide gel and concentrated by ethanol precipitation. The protein solution was mixed with labelled DNA at different concentrations of the protein for 30 min in 20 m*M* Tris, 200 m*M* NaCl pH 7.5 in a total volume of 20 µl before adding 0.2 µl DNase I. The reaction was stopped by the addition of 12.5 µl 3 *M* ammonium acetate, 0.25 *M* EDTA. The DNA was ethanol precipitated (3 µl of 3 mg ml^−1^ yeast tRNA was added to the precipitation solution for higher DNA recovery) and analysed on a 6% denaturing polyacrylamide gel using formamide dye (0.03% xylene cyanol, 0.03% bromophenol blue, 20 m*M* EDTA dissolved in formamide) as a loading buffer. Reference sequencing ladders were prepared from the same ^32^P-ATP labelled DNA using citrate or hydrazine following the Maxam–Gilbert sequencing method (Maxam & Gilbert, 1980 ▸).

### 2.10. Small-angle X-ray scattering

Small-angle X-ray scattering (SAXS) data for YdaT and its mutants were collected in HPLC mode on the SWING beamline at the SOLEIL synchrotron, Gif-sur-Yvette, France. The data for YdaT in complex with the O_M_ operator fragment were collected on beamline BM29 at the European Synchrotron Radiation Facility (ESRF), Grenoble, France, also in HPLC mode. Details of data collection and analysis are given in Supplementary Table S4. Samples were dialysed against 20 m*M* Tris–HCl, 200 m*M* NaCl pH 8 with three buffer changes, with the last buffer change left to dialyse overnight. The dialysis buffer was filtered through a 0.20 µm HAWP filter. For free YdaT and its mutants, a Shodex KW402.5-4F column (Showa Denko K. K.) was equilibrated with the dialysis buffer and 45 µl sample at 10 mg ml^−1^ was injected into the column. The sample was run at 0.3 ml min^−1^. Prior to measurements, the sample was centrifuged at 13 300 rev min^−1^ for 10 min. The YdaT–DNA complex for SAXS measurements was prepared by mixing YdaT with an excess of the oligonucleotide O_M_. The complex was left to incubate for 30 min and then injected onto an ENrich SEC 650 column (Bio-Rad). The peak corresponding to the complex was collected and concentrated to a final volume of 50 µl. The complex was then injected onto a pre-equilibrated AdvanceBio SEC 300 column (Agilent) and run at 0.16 ml min^−1^ at the beamline. Radiation damage was monitored by evaluating *R*_g_ values per frame during data collection. The data were normalized to the intensity of the transmitted beam and radially averaged. The contributions of the buffer to the scattering were measured at the beginning of the elution and were subtracted from the scattering of the protein. The data from the SWING beamline were processed with *Foxtrot* (David & Pérez, 2009 ▸) and those from the BM29 beamline were processed with *CHROMIXS* (Panjkovich & Svergun, 2018 ▸).

All simulations were performed in *XPLOR-NIH* version 2.49 (Schwieters *et al.*, 2003 ▸). Refinement of the YdaT tetramer against the experimental SAXS data was carried out starting from the crystal structure presented in this work. Protons and atoms of the residues that were not resolved in the X-ray structure were added in *XPLOR-NIH*, followed by minimization of the energy function consisting of the standard geometric (bonds, angles, dihedrals and impropers) and steric (van der Waals) terms. The position of the C-terminal four-helix bundle was kept fixed and the N-terminal POU domains were treated as rigid-body groups, while the N- and C-terminal tails and the flexible hinges (residues Ser96–Tyr99) were given full torsional degrees of freedom. YdaT^1–96^ was refined as a monomer. The POU domain was kept fixed, while the N-terminal purification tag of YdaT^1–96^ was allowed to move. The computational protocol comprised an initial simulated-annealing step followed by side-chain energy minimization as described previously (Schwieters *et al.*, 2003 ▸; Schwieters & Clore, 2014 ▸). In addition to the standard geometric and steric terms, the energy function included a knowledge-based dihedral angle potential and a SAXS energy term incorporating the experimental data (Schwieters & Clore, 2014 ▸; Schwieters *et al.*, 2018 ▸).

For refinement of the YdaT–DNA complex, the atomic coordinates were taken from a model built from the crystal structure of YdaT solved in this work (PDB entry 8bt1) and that of mouse Oct-4 bound to its operator DNA (PDB entry 6ht5; J. Vahokoski, V. Pogenberg & M. Wilmanns, unpublished work). The position of the C-terminal four-helix bundle was kept fixed and pairs of YdaT DNA-binding domains and their respective DNA double-stranded helix were grouped as rigid-body units, while the N- and C-terminal tails and the flexible hinge (residues Ser96–Tyr99) were given full torsional degrees of freedom. Multiple copies of the molecular system (*N* = 1–5) were refined simultaneously in order to simulate molecular ensembles of multiple conformers (Schwieters & Clore, 2014 ▸).

In each refinement run, 100 structures were calculated and the ten lowest-energy solutions, representing the best agreement with the experimental data, were retained for subsequent analysis. The agreement between the experimental and calculated SAXS curves (obtained with the *calcSAXS* helper program, which is part of the *XPLOR-NIH* package) was assessed by calculating χ^2^, 

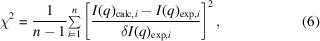

where *I*(*q*)_calc,*i*_ and *I*(*q*)_exp,*i*_ are the scattering intensities at a given *q* for the calculated and experimental SAXS curves, respectively, δ*I*(*q*)_exp,*i*_ is the experimental error on the corresponding *I*(*q*)_exp,*i*_ value and *n* is the number of data points defining the experimental SAXS curve.

## 3. Results

### 3.1. YdaT belongs to the POU family

YdaT was crystallized and its structure was determined at 2.4 Å resolution to *R*_work_ = 0.195 and *R*_free_ = 0.269 (Table 2 ▸ and Supplementary Tables S2 and S3). The crystal contained a tetramer in the asymmetric unit. The X-ray data are highly anisotropic, and despite anisotropy correction using *STARANISO* the *R* factors remained relatively high. While stereochemical parameters and density fit are good for chains *A* and *B*, they are significantly poorer for chain *C* and especially for chain *D*, despite careful model building combined with experimenting with different refinement strategies. The final structure was built for residues Lys2–Leu124. The N-terminal His tag as well as the C-terminal residues Tyr125–His141 are disordered and do not provide interpretable electron density.

**Figure 2:**
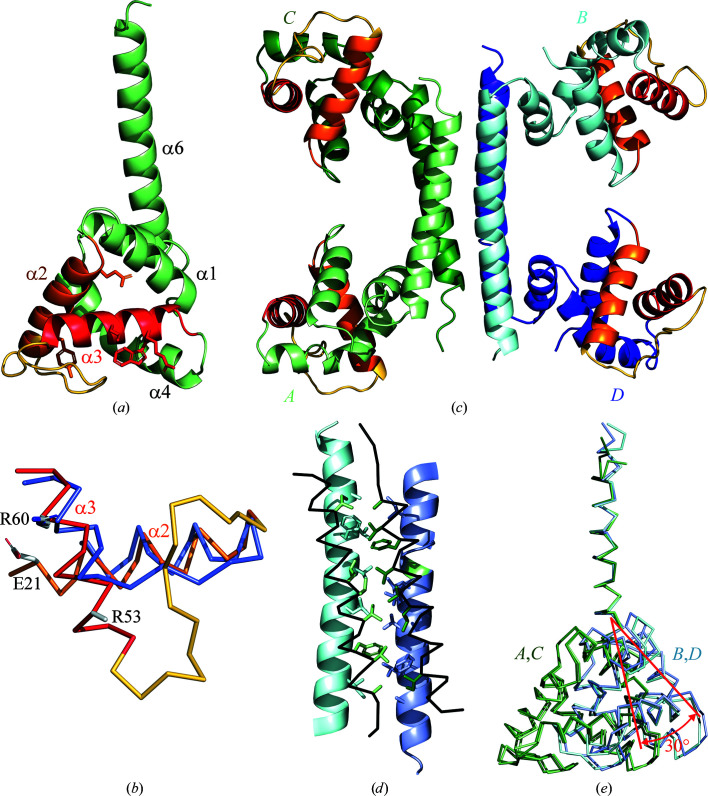
Structure of YdaT. (*a*) Cartoon representation of the YdaT monomer (chain *A* from PDB entry 8bt1, this work). Secondary-structure elements are labelled except for helix α5, which is hidden behind helices α2 and α3. The helix–turn–helix motif is highlighted, with the recognition helix (α3) in red, helix α2 in orange and the connecting loop in yellow. (*b*) Cartoon representation of the YdaT tetramer with each chain in a distinct colour. The two subunits in shades of green form one functional DNA-binding dimer (chains *A* and *C* in PDB entry 8bt1), while the other two subunits in shades of blue (chains *B* and *D*) form the second functional DNA-binding dimer. (*c*) Superposition of the HTH motifs of YdaT [coloured as in (*a*)] and mouse Oct-4 (blue; PDB entry 6ht5) based on their POU domains (Phillips & Luisi, 2000 ▸). The long loop between helices α2 and α3 is absent from Oct-4 and the recognition helix α3 is significantly longer in YdaT compared with Oct-4. (*d*) Tetramer formation though the creation of a four-helix bundle. In two subunits helix α6 is shown as a cartoon, while in the other two subunits this helix is shown as a grey C^α^ trace. Side chains that make up the hydrophobic core are shown as sticks. (*e*) Superposition of the four subunits in the crystal structure of the YdaT tetramer. The tetramerization helices α6 are superimposed, showing the variability in orientation of the corresponding POU domains. The POU domains of chains *A* and *C* are coloured green and oriented differently from the POU domains in chains *B* and *D*, which are coloured blue.

The YdaT monomer consists of an N-terminal globular helix–turn–helix (HTH)-containing domain followed by a long 29-residue α-helix (Fig. 2 ▸ *a* and Supplementary Fig. S1*a* ). The globular domain is all-α, with four longer four- to five-turn α-helices (α1–α4 in Fig. 2 ▸ *a* and Supplementary Fig. S1*a* ) followed by a shorter two-turn α-helix (α5). A *DALI* search with residues Lys4–Met82 of the YdaT monomer picks up a whole series of DNA-binding proteins that all contain a helix–turn–helix structural motif. Next to the obvious YdaT from *E. coli* O6:H1 (PDB entry 3c4r; *Z*-score of 20.1 and 84% sequence identity), the best matches involve POU-domain transcription factors (Table 3 ▸). POU domains are conserved domains that are found in eukaryotes, with the acronym referring to three transcription factors (Pit-1, Oct1/2 and Unc-86) in which the domain was first described (Phillips & Luisi, 2000 ▸). The first bacterial match is the transcription regulator ClgR from *Corynebacterium glutamicum* (Russo *et al.*, 2009 ▸), while the first toxin–antitoxin-related match is HipB from *Shewanella oneidensis* (Wen *et al.*, 2014 ▸).

A long (14-residue) loop between helices α2 and α3 making up the HTH motif is unique to YdaT and is absent from all other POU-domain structures. In addition, the central α-helix α3 (Ala50–Asp63, which is the recognition helix in the HTH motif) of YdaT is longer than the corresponding helix in POU-domain structures such as Pit-1 or Oct-4 (PDB entries 1au7 and 6ht5), and α-helix α1 (Glu6-His17) is in a different relative orientation (Figs. 2 ▸ *a* and 2 ▸ *b*). A *BLAST* search (Altschul *et al.*, 1997 ▸) of our YdaT sequence against the UniProtKB reference proteomes and Swiss-Prot database resulted in 40 hits with an *E*-value smaller than 0.1. In these sequences the α2–α3 loop varies in length between nine and 23 residues and does not contain any obviously conserved residues (Supplementary Fig. S1*b* ).

The POU domain of YdaT is highly rigid, with r.m.s.d. values between 0.4 and 0.5 Å for all backbone (N, C^α^, C and O) atoms of residues 3–90. Only Asp44–Glu49, which corresponds to the C-terminal part of the long YdaT-specific loop between helices α2 and α3, shows some small variation.

### 3.2. YdaT is a tetramer in solution

YdaT forms a tetramer in the crystal (Fig. 2 ▸ *c*). The long C-terminal α-helix α6 serves as an oligomerization motif, with four such helices assembling into an antiparallel bundle, burying a total surface of around 2600 Å^2^. A hydrophobic core is formed via the side chains of Ile103, Leu110, Val111, Val114, Phe117, Val118 and Ala121 (Fig. 2 ▸ *d*). This C-terminal α-helix α6 can bend slightly, changing the direction of its N-terminus. Chains *A* and *C* adopt very similar conformations, as do chains *B* and *D*. When both pairs are compared and superimposed using the C-terminal helix α6 (residues Tyr100–Ser120), the orientation of the N-terminal domain rotates by almost 30° (Fig. 2 ▸ *e*). The loop Ser94–Tyr99 here serves as a hinge region. The POU-like DNA-binding domains themselves are highly similar, with only some small variations in backbone structure in the loop between helices α2 and α3.

**Figure 3:**
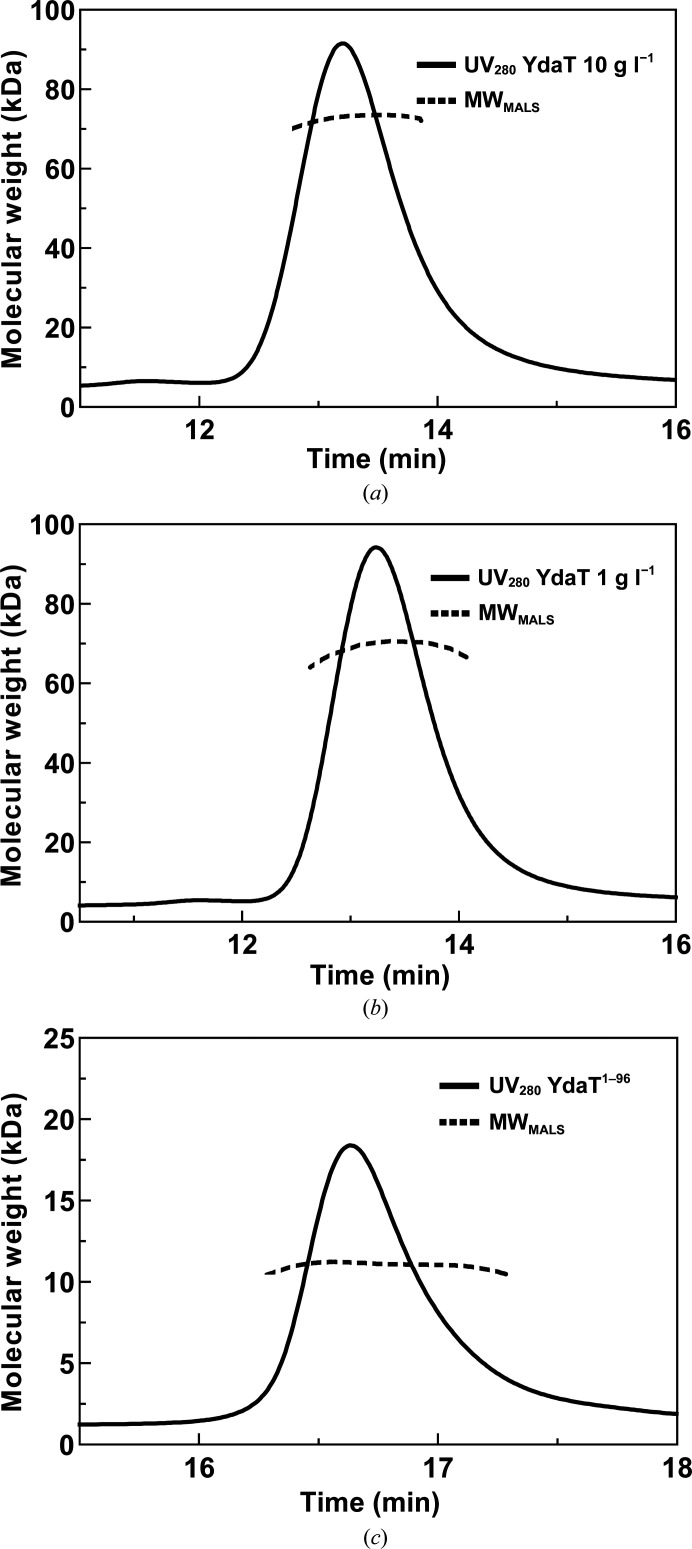
SEC-MALS. (*a*) SEC-MALS profile of YdaT at a concentration of 10 mg ml^−1^. (*b*) A similar profile but for a concentration of 1 mg ml^−1^. The molecular weight determined from both concentrations is very similar (72.9 and 69.4 kDa) and is within the margin of error of the technique. (*c*) SEC-MALS profile of YdaT^1–96^ at a concentration of 10 mg ml^−1^. With an experimental molecular weight of 11.1 kDa, this corresponds to a monomer.

**Figure 4:**
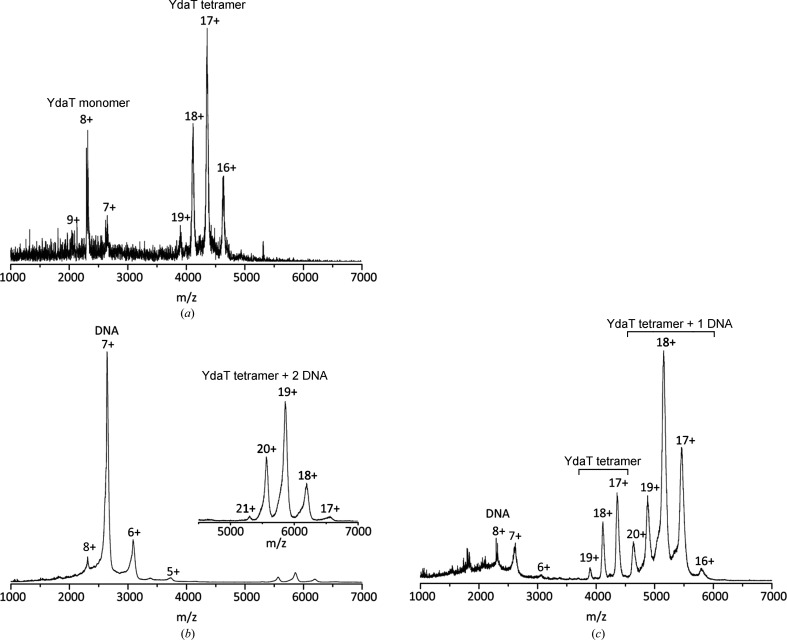
Native mass spectrometry. (*a*) Native MS spectrum of YdaT at a concentration of 2.5 µ*M* (tetramer concentration). Next to the expected tetramer, peaks corresponding to a YdaT monomer are also visible. (*b*) When an excess amount of the 30 bp O_M_ operator fragment (15 µ*M* of duplex) is added to YdaT (2.5 µ*M* of tetramer), a single species of a YdaT tetramer bound to two O_M_ duplexes is observed next to an excess of free DNA. (*c*) When the O_M_ DNA duplex (1.67 µ*M*) becomes saturated with YdaT (2.5 µ*M*), the dominant species becomes a YdaT tetramer bound to a single O_M_ duplex.

To determine the oligomeric state of YdaT in solution, we performed SEC-MALS. The elution profile (injected monomer concentrations of 54.1 and 541.4 µ*M*, corresponding to 1 and 10 mg ml^−1^, respectively) shows a single symmetrical peak corresponding to molecular weights of 69.4 or 72.9 kDa, respectively, in close agreement with the theoretical mass of 73.9 kDa for a tetramer (Figs. 3 ▸ *a* and 3 ▸ *b*). Native mass-spectrometry measurements confirm this result. At a concentration of 1.0 µ*M* YdaT (monomer equivalents) is primarily a tetramer, but some monomer is also present (Fig. 4 ▸ *a*).

**Figure 5:**
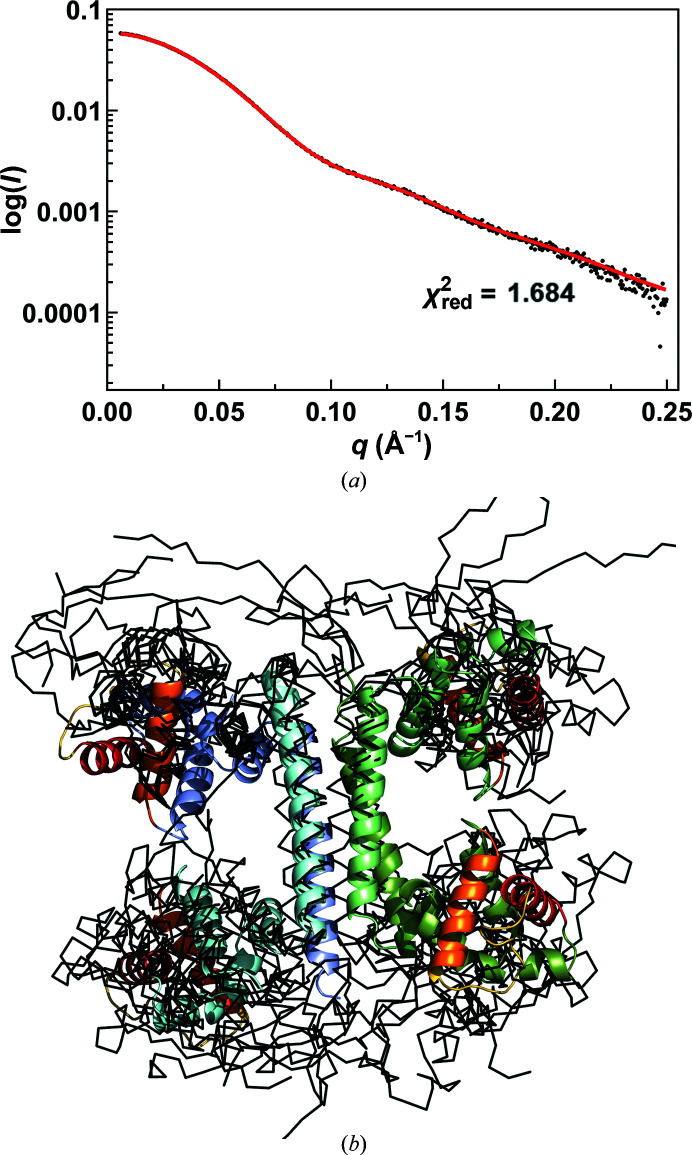
SAXS solution structure of YdaT. (*a*) Experimental SAXS data of YdaT (black) and the calculated curve (red) obtained from the best-fitting ensemble of ten conformers (χ^2^ = 1.684). (*b*) Superimposition of the ten conformers of the YdaT SAXS ensemble (black C^α^ traces) onto the YdaT crystal structure (cartoon representation coloured as in Fig. 2 ▸ *b*). In the SAXS ensemble, the POU domains adopt different orientations relative to the C-terminal four-helix bundle due to the flexibility of the loop between the C- and N-terminal domains (residues 96–99).

The tetramer observed in our crystal structure does not perfectly match 222 symmetry. Because of differences in bending and hinge conformation, the POU domains in chains *A* and *C* are significantly further from each other than those in chains *B* and *D* (10.9 versus 5.1 Å as their closest distance). Similar relative movements of the POU domains are also observed in the structure of the closely related uncharacterized protein in PDB entry 3c4r. In order to further characterize these inter-domain dynamics in solution, we performed SEC-SAXS (Figs. 5 ▸ *a* and 5 ▸ *b*). A good fit of the data (χ^2^ = 1.68) is obtained with an ensemble of ten structures where the N-terminal domains are allowed to move relative to the C-terminal four-helix bundle, confirming the structural variability observed in the crystal.

### 3.3. YdaT recognizes an inverted repeat located between the *ydaS* and *ydaT* genes

**Figure 6:**
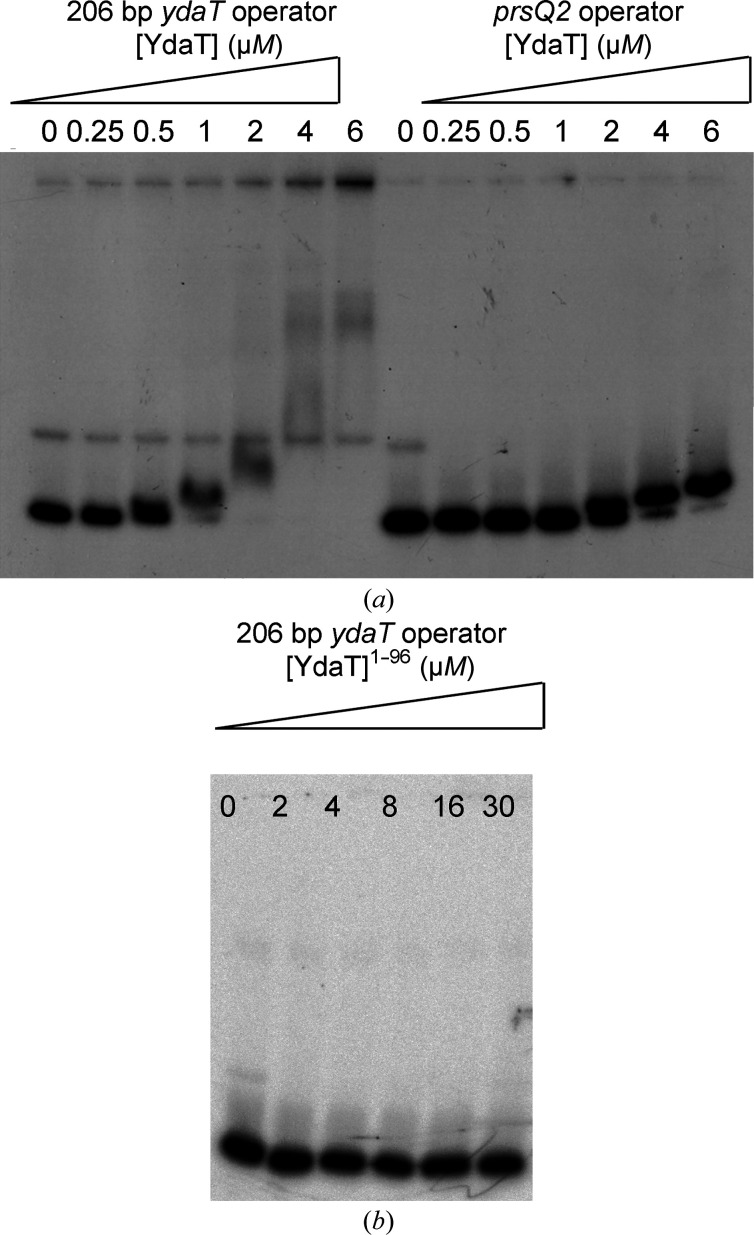
EMSA. (*a*) EMSA experiments performed using increasing concentrations of YdaT tetramers on either a 206 bp fragment of the *ydaT* promotor/operator region or a similar sized fragment of the *Cupriavidus metallidurans prsQ2* promotor/operator region. (*b*) EMSA of YdaT^1–96^ on the 206 bp *ydaT* promotor/operator DNA fragment showing a lack of binding.

The structural organization of the prophage CP-933P suggests YdaS and YdaT as the equivalents of the Cro and CII repressors in λ, a hypothesis that was recently substantiated by *in vivo* experiments (Jurėnas *et al.*, 2021 ▸). As CII binds to the *P_RE_* promoter located between the *cro* and *cII* genes in λ, we tested *in vitro* whether YdaT can interact with a 206 bp segment covering the end of the *ydaS* gene and the start of the *ydaT* gene. EMSA experiments show concentration-dependent binding to this fragment (Fig. 6 ▸ *a*). Several bands can be observed with increasing YdaT concentration, indicating multiple binding events. In contrast, binding to the unrelated promotor/operon region of *prsQ*2 from *Cupriavidus metallidurans* NA4 requires roughly fourfold higher YdaT concentrations and does not lead to the observation of multiple species.

**Figure 7:**
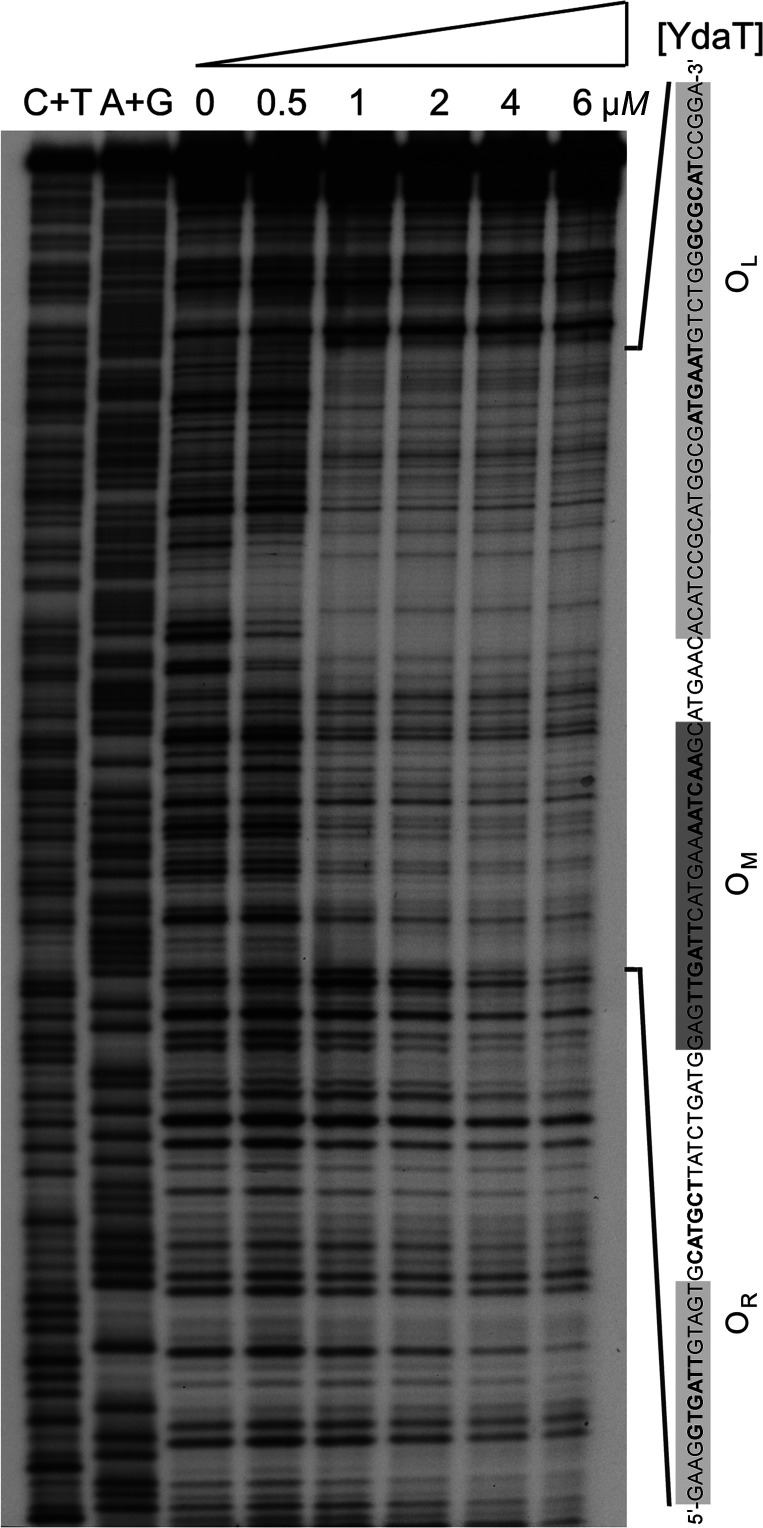
DNAse I footprinting. DNase I footprint on a 236 bp operator fragment (bottom strand shown) using increasing concentrations of YdaT (in tetramer equivalents). The nucleotide sequence of the region that becomes protected is shown at the side with an indication of the regions of protection. The sequence of the 95 nt protected region is indicated on the right. At 0.5 µ*M* YdaT, a 25 bp zone of protection (dark grey background) containing the inverted repeat 5′-TTGATTN_6_AATCAA-3′ (bold) can already be observed. At higher protein concentrations this zone is extended further on both sides of the primary binding site, resulting in a total footprint of 94–97 nt on both strands that consists of zones of stronger (light grey background) and weaker (white background) protection. The former overlap with two imperfect inverted repeats (bold). These inverted repeats are labelled O_L_, O_M_ and O_R_ as described in the text.

In order to pinpoint the exact binding site of YdaT, we turned to DNase I footprinting using a slightly longer 236 bp fragment (Fig. 7 ▸). At the lowest protein concentration used (0.5 µ*M*), protection was already observed on both strands for a 25 bp region containing a 5′-TTGATTN_6_AATCAA-3′ inverted repeat located at the end of the coding region of the *ydaS* gene and downstream from the *P_RE933P_* promotor that was proposed as an alternative transcription start for the *paaR2–paaA2–parE2* operon and which is controlled via YdaT (Jurėnas *et al.*, 2021 ▸). This highly protected inverted repeat is referred to as O_M_ (Figs. 1 ▸ and 7 ▸). At higher protein concentrations, starting from 1.0 µ*M*, this DNase I-protected area extends further on each side, resulting in a total region of protection of approximately 95 nt on both strands that is composed of zones of weaker and stronger protection. The latter contain sequences that show sequence similarity to part of the high-affinity binding site and may represent additional lower affinity binding sites, thus explaining the multiple bands observed in the EMSA experiments. These two incomplete inverted repeats are referred to as O_L_ and O_R_ (Figs. 1 ▸ and 7 ▸).

### 3.4. Thermodynamics of operator binding

**Figure 8:**
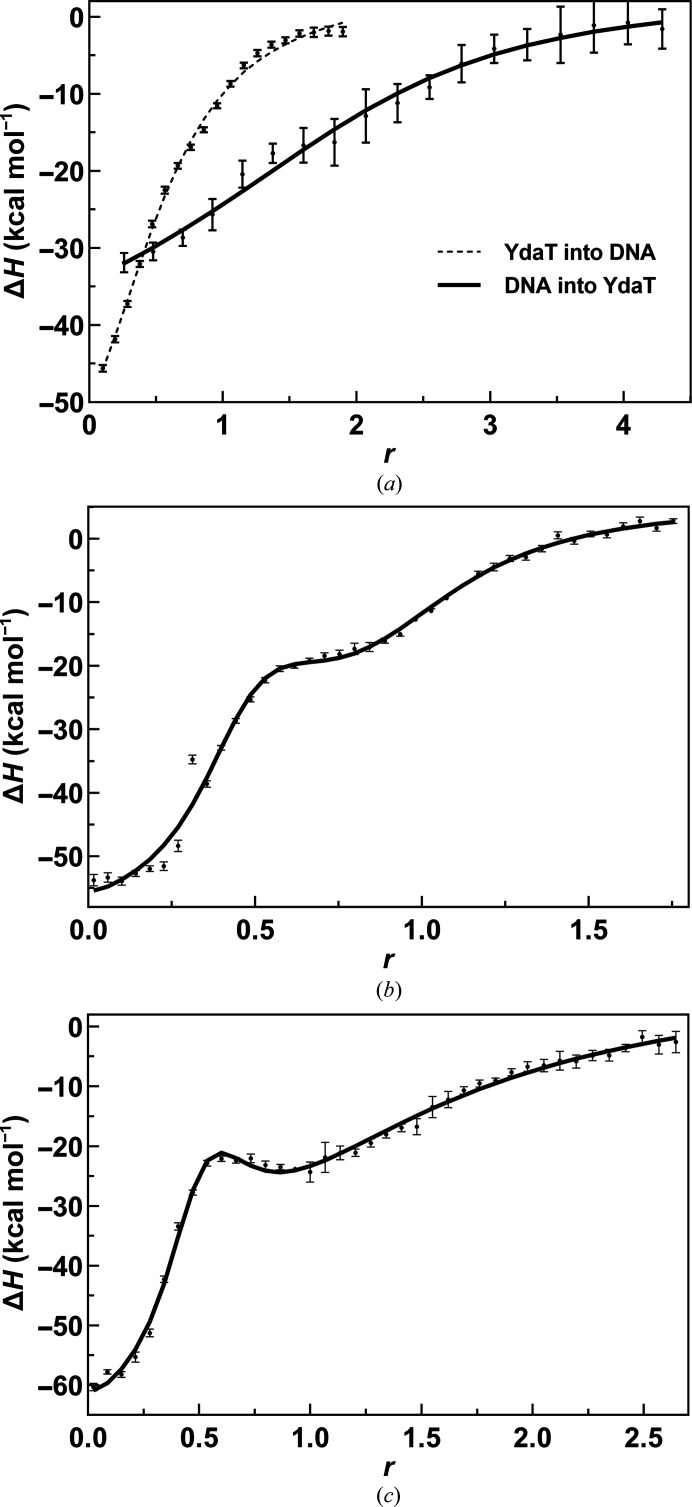
Isothermal titration calorimetry. (*a*) Binding between YdaT and O_M_. The integrated heat of binding per mole of injectant is plotted as Δ*H* as a function of the molar ratio *r* between the host and the ligand in the cell and the syringe, respectively, and fitted as described in Section 2. The full line corresponds to a titration with 5 µ*M* YdaT tetramer in the cell titrated with 100 µ*M* O_M_ duplex in the syringe. The dashed curve corresponds to a titration with 5 µ*M* O_M_ duplex in the cell and 25 µ*M* YdaT tetramer in the syringe. A single global fit for titrations was used to obtain the thermodynamic parameters listed in Table 4 ▸. (*b*) A similar titration for YdaT (100 µ*M* tetramer) titrated into to the O_MR_ duplex (15 µ*M*). (*c*) A similar titration for YdaT tetramer (140 µ*M*) titrated into the O_LM_ duplex (15 µ*M*). All molar ratios (*r* = [syringe]/[cell]) were calculated for YdaT as a tetramer and the DNA fragment as a duplex.

Next, we turned to isothermal titration calorimetry (ITC) to understand how YdaT binds to its operator region. We therefore selected a set of fragments containing different potential binding sites (Table 4 ▸, Supplementary Fig. S5). Fragment O_M_ contains the central inverted repeat that was identified as the main binding site for YdaT in the DNase I protection experiment. This fragment binds to YdaT with an affinity in the submicromolar range and two binding events can be discerned that differ in affinity by approximately a factor of four (Table 4 ▸, Fig. 8 ▸, Supplementary Fig. S5). This indicates that the YdaT tetramer binds two duplexes of O_M_ with apparent negative cooperativity of enthalpic origin.

To further confirm this proposed binding model to O_M_, we turned to native mass spectrometry (MS). With an excess of O_M_, all YdaT tetramers are saturated with two such DNA fragments, leading to a species of 110.7 kDa. When YdaT is in excess, on the other hand, YdaT tetramers with both one and two O_M_ fragments bound are observed (Figs. 4 ▸ *b* and 4 ▸ *c*, Supplementary Fig. S3), in agreement with the results from ITC.

In contrast, fragments containing the possible alternative incomplete inverted-repeat sequences O_L_ or O_R_ did not allow high-quality ITC data to be measured. Both fragments bind only weakly and no reliable thermodynamic parameters could be obtained (Supplementary Fig. S2). Similarly, longer fragments combining O_M_ with O_L_ or O_R_ (O_LM_ and O_MR_) show a combination of the two high-affinity events seen for O_M_ as well as a lower affinity event (Table 4 ▸, Fig. 8 ▸). Binding to these low-affinity sites increases the affinity for the main site by a factor of 2–4, indicating an interaction between the different sites. Given the distance between these sites, especially in the case of O_LM_, this communication is likely to propagate through the DNA rather than through direct contacts between two bound YdaT tetramers.

### 3.5. SAXS model of the YdaT–operator complex

The four DNA-binding domains of YdaT are oriented such that two surfaces are generated that face away from each other, and in principle each can accommodate an ∼30 bp DNA duplex. This is in agreement with the binding stoichiometry that is obtained from ITC and native MS. Using the structure of mouse octamer-binding protein 4 (Oct-4) in complex with a 21 bp DNA duplex (PDB entry 6ht5) as a guide, we built a model of YdaT bound to a 30 bp B-DNA duplex containing the O_M_ sequence identical to that used for ITC (O_M_ in Supplementary Table S4).

**Figure 9:**
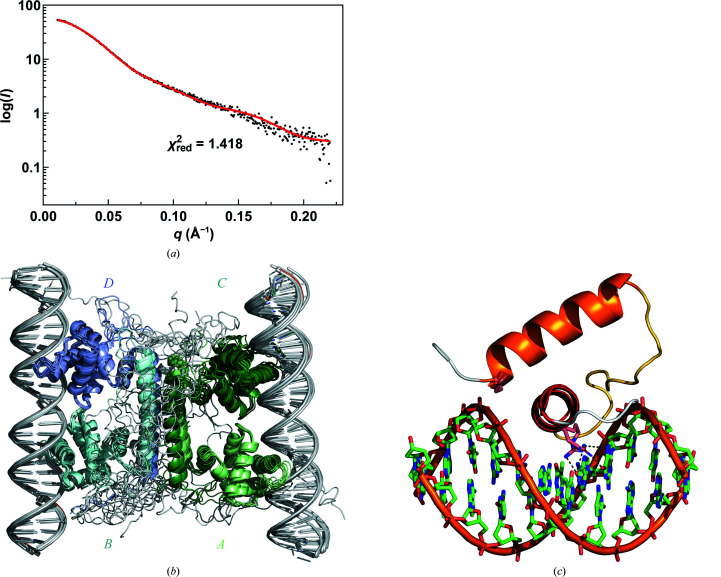
SAXS solution structure of the YdaT–O_M_ complex. (*a*) Experimental SAXS curve (black) with the theoretical curve for YdaT–O_M_ (red) superimposed. The theoretical curve was calculated from the ten best-fitting ensembles (χ^2^ = 1.418) each consisting of two models. (*b*) YdaT tetramer bound to two O_M_ duplexes. Ten superimposed conformers from the SAXS ensembles are shown. The DNA–POU-domain assemblies show rigid-body movement relative to the four-helix core of the YdaT tetramer. Within the POU domains, structural variations relative to the free structure are limited to the N-terminal His tag and the C-terminal tail as well as the loop (residues 96–99) between the POU domain and C-terminal helix α6. (*c*) Enlargement of the HTH motif bound to the O_M_ duplex.

Next, this model was validated using SAXS (Fig. 9 ▸ and Supplementary Table S4). The molecular weight determined from the SAXS data is about 102 kDa, which is close to the theoretical molecular weight of 110.7 kDa for a complex consisting of one YdaT tetramer and two O_M_ molecules. The central four-helix bundle was fixed, while the POU domains were allowed to reorient while remaining docked onto the DNA through variation of the hinge loop Ser96–Tyr99. The N-terminal His tag and the C-terminal 20 amino acids remain highly flexible. As a consequence, pairs of two POU domains (chains *A* and *C* or chains *B* and *D*) remain locked together via the bridging DNA molecule and their movements relative to the C-terminal helix α6 become highly restricted compared with those observed in the free structure (Figs. 9 ▸ *a* and 9 ▸ *b*). The best fit to the experimental data (χ^2^ = 1.418) was obtained with ten ensembles each containing two conformers. More conformers did not improve the fit.

Compared with the crystal structure of the free form of YdaT, no conformational changes are required in YdaT except for some rigid-body movement of the different POU domains relative to each other and the reorientation of a few side chains. In particular, the *A*–*C* pair of POU domains are oriented in our crystal structure of the free state such that they cannot correctly bind together to the same DNA duplex.

The residues of the Leu35–Glu49 loop fold over the ribose-phosphate backbone of one DNA strand, while the recognition helix α3 (Ala50–Asp63) of the HTH motif docks into the major groove of the DNA (Fig. 9 ▸ *c*). Arg60 is nicely positioned to make base-specific hydrogen bonds to Gua21. This arginine is highly conserved in all available POU-domain structures, where it makes similar contacts. It is also conserved in all sequences of YdaT homologs picked up by our *BLAST* search. Interestingly, in some of these sequences an insertion of a single amino acid between Phe59 and Arg60 is observed that is predicted by *AlphaFold*2 to result in a single turn of π-helix to allow the side chain of Arg60 to remain in the correct orientation and position. The side chain of Arg53 is likely to form a hydrogen bond to the neighbouring backbone phosphate group of Thy10. Residues of the His17–Gly20 loop together with the N-terminus of helix α2 (Glu21–Glu34) are in contact with the other DNA strand and a hydrogen bond between the backbone amide of Glu21 and an O atom from the ribose backbone is likely. This residue is also not conserved in the eukaryotic POU domains or in the bacterial YdaT homologs.

### 3.6. Oligomerization of YdaT is required for DNA binding

In order to understand the role of oligomerization in DNA binding, we created a mutant in which YdaT is truncated after His96 (YdaT^1–96^). This corresponds to a protein consisting of the N-terminal domain but lacking the C-terminal oligomerization helix α6. YdaT^1–96^ appears to be a well folded species in solution with a predominantly α-helical structure (Supplementary Fig. S4*a* ), in agreement with the conformation of this domain in the crystal structure of the full-length protein. SEC-MALS and SAXS further support this conclusion (Supplementary Table S4, Fig. 3 ▸ *c*, Supplementary Figs. S5*a* and S5*b* ). No measurable interaction between YdaT^1–96^ and the YdaT operator DNA was detected in EMSA experiments (Fig. 6 ▸ *b*), indicating that a monomeric POU domain is insufficient for effective DNA binding. The molecular weights determined from SEC-MALS and SAXS analysis are 11.1 and ∼12 kDa, respectively, and are in close agreement with the theoretical molecular weight of 13.3 kDa for a monomeric species. The theoretical scattering curve calculated for the ensemble of ten conformers fits the experimental curve well (χ^2^ = 0.983).

## 4. Discussion

YdaT was originally identified as one of two potential transcripts encoded by the *ydaST* operon in *E. coli* OH157:H7. This operon was originally suspected to encode a toxin–antitoxin pair (Sevin & Barloy-Hubler, 2007 ▸), but was recently shown to be part of the cryptic prophage CP-933P, where the cognate proteins function as equivalents of λ Cro and CII (Jurėnas *et al.*, 2021 ▸). The cryptic prophage CP-933P has lost its ability to enter a lytic cycle. Its immunity region contains the *paaA2–parE2* toxin–antitoxin gene pair that replaces the *rexA* and *rexB* genes and is preceded by the gene for the PaaR2 regulator that replaces λ CI.

CP-933P YdaT is a representative of a family of transcription regulators found in lambdoid phages and is functionally but not structurally related to λ CII. The DNA-binding domain of YdaT is a POU domain, with an unusually long loop between the two helices of the HTH motif (α2 and α3) as a defining structural feature. A sequence alignment of YdaT homologs shows that this long loop varies in length and sequence within the YdaT family (Supplementary Fig. S1*b* ) and does not contain obvious conserved residues, despite being likely involved in operator binding. Equally, the recognition helix α3 contains only a single residue that is fully conserved in the YdaT family, Arg60, which according to our model is likely to be essential for operator binding. The evolutionary pressure that drives this variability even though the protein is still functional as a repressor in a cryptic phage such as CP-933P (Jurėnas *et al.*, 2021 ▸) is unclear. It is possible that it relates to a requirement to stably maintain cryptic prophages or segments thereof in the genome, similar to toxin–antitoxin pairs of the same family that are only found together on the same chromosome if they do not interact (Goeders & Van Melderen, 2014 ▸ and references therein).

YdaT of the cryptic prophage CP-933P is functional as a DNA-binding protein, as illustrated by our EMSA, ITC and DNAse I protection experiments as well as by previously reported *in vivo* data (Jurėnas *et al.*, 2021 ▸). This functionality requires oligomerization. YdaT is a symmetric tetramer with two oppositely positioned sets of DNA-binding sites, meaning that it can recognize two 30 bp operator sequences simultaneously. Yet, the CP-933P prophage only contains a single strong binding site, possibly leaving one pair of POU domains unbound. Alternatively, YdaT has the potential to stabilize a DNA loop. However, the distance between the strong main binding site and the two flanking potential secondary sites is too short to allow the formation of a loop. Indeed, weak binding to these sites seems to occur independent of binding to the main site.

CII, the equivalent of YdaT in λ, is also a tetramer, but rather than forming a closed point group has an ‘unusual dimer-of-dimers’ architecture in which two of the subunits (each from a different dimer) are correctly positioned relative to each other to recognize a direct repeat on the operator (Datta *et al.*, 2005 ▸; Jain *et al.*, 2005 ▸). The other two monomers form a bridge between the ‘active’ subunits but are not themselves involved in DNA binding. In this architecture the two DNA-binding subunits are oriented in the same direction, as would be required for recognition of a direct repeat. YdaT, on the other hand, forms a more classic type of tetramer with internal 222 point-group symmetry and is therefore suited to recognize an inverted repeat.

## Supplementary Material

PDB reference: YdaT, 8bt1

Supplementary Tables and Figures. DOI: 10.1107/S2059798323001249/nz5012sup1.pdf

## Figures and Tables

**Table 1:** Macromolecule-production information

Protein	Wild-type YdaT	YdaT^1–96^
Source organism	*Escherichia coli* O157:H7	*Escherichia coli* O157:H7
DNA source	Commercial gene synthesis by GenScript	Commercial gene synthesis by GenScript
Cloning vector	pET-28b	pET-28b
Expression vector	pET-28b	pET-28b
Expression host	*E. coli* BL21 (DE3)	*E. coli* BL21 (DE3)
Complete amino-acid sequence of the construct produced	MGSSHHHHHHSSGENLYFQGASKIKHEHIRMAMNVWAHPDGEKVPAAKITKAYFELGMTFPELYDDSHPEALARNTQKIFRWLDKDTPDAVEKMQALLPAIEKAMPPLLVARMRSHSSEYYREIVERRDRLVKDVDDFVASAVVLYDQMNRGGPAGNAVVMH	MGSSHHHHHHSSGENLYFQGASKIKHEHIRMAMNVWAHPDGEKVPAAKITKAYFELGMTFPELYDDSHPEALARNTQKIFRWLDKDTPDAVEKMQALLPAIEKAMPPLLVARMRSHS
Molecular mass from chemical composition	18472.02	13348.27
Extinction coefficient (*M* ^−1^ cm^−1^)	19940	15470

**Table 2:** Crystallographic data collection and refinement Values in parentheses are for the outer shell. *XDS* was used for data collection and scaling.

Space group	*P*1
*a*, *b*, *c* (Å)	45.57, 64.26, 63.91
α, β, γ (°)	66.83, 89.36, 77.67
Resolution range (Å)	44.37–2.40 (2.52–2.40)
Completeness (spherical) (%)	77.4 (32.5)
*R*_merge_	0.082 (0.386)
*R*_meas_	0.106 (0.552)
CC_1/2_	0.990 (0.797)
No. of reflections, working set	18719
No. of reflections, test set	979
Final *R*_cryst_	0.2112 (0.2757)
Final *R*_free_	0.2658 (0.2783)
R.m.s. deviations	
Bond lengths (Å)	0.0088
Bond angles (°)	0.959
Ramachandran plot	
Most favoured (%)	97.4
Additionally allowed (%)	2.0
Disallowed (%)	0.6
PDB code	8bt1

**Table 3:** Structural homologs of the N-terminal domain of YdaT picked up in a *DALI* search The list shows the ten closest structures after removing duplicates.

Protein	Organism	PDB entry, chain	*DALI Z*-score	R.m.s.d. (Å)	Sequence identity† (%)	No. of common C^α^ atoms	Reference
YdaT	*Escherichia coli* O6	3c4r, *A*	20.1	0.3	84	82	New York SGX Research Center for Structural Genomics (unpublished work)
Pit-1	*Rattus norvegicus*	1au7, *B*	4.9	3.0	11	61	Jacobson *et al.* (1997 ▸)
Oct-4	*Homo sapiens*	6yov, *K*	4.9	2.1	11	57	Michael *et al.* (2020 ▸)
Oct-1	*Homo sapiens*	1hf0, *A*	4.8	3.0	10	68	Reményi *et al.* (2001 ▸)
Oct-4	*Mus musculus*	6ht5, *E*	4.5	2.5	11	57	J. Vahokoski, V. Pogenberg & M. Wilmanns (unpublished work)
Oct-6	*Mus musculus*	2xsd, *C*	4.4	3.1	15	62	Jauch *et al.* (2011 ▸)
Pit-1	*Homo sapiens*	5wc9, *A*	4.4	3.0	10	62	Agarwal & Cho (2018 ▸)
Brn-5	*Homo sapiens*	3d1n, *P*	4.1	2.9	10	60	Pereira & Kim (2009 ▸)
ClgR	*Corynebacterium glutamicum*	3f51, *C*	4.0	2.4	10	50	Russo *et al.* (2009 ▸)
HipB	*Shewanella oneidensis*	4pu7, *B*	3.6	2.5	8	52	Wen *et al.* (2014 ▸)

† N-terminal domain, residues 1–96.

**Table 4:** Thermodynamics of operator binding obtained from ITC The *K*_d_ and Δ*H* parameters were obtained from fitting the model equations to the ITC data and are reported at *T* = 298 K. In all cases index 1 refers to the binding of the central inverted repeat to the first YdaT binding site and index 2 refers to the binding of the same repeat from another DNA molecule to the second YdaT binding site. Index 3 refers to the binding of YdaT to the distant incomplete repeat (R or L), but it is not clear whether one or two YdaT binding sites are engaged in binding. The free energy of association Δ*G* and the entropic contribution *T*Δ*S* were calculated using standard equations. Standard mean errors are obtained from the fitting procedure or are calculated through error propagation in the case of *T*Δ*S* and Δ*G*. *K*_d_ values are given in µ*M*, while Δ*G*, Δ*H* and *T*Δ*S* values are in kcal mol^−1^.

DNA	*K*_d1_	Δ*G*_1_	Δ*H*_1_	*T*Δ*S*_1_	*K*_d2_	Δ*G*_2_	Δ*H*_2_	*T*Δ*S*_2_	*K*_d3_	Δ*G*_3_	Δ*H*_3_	*T*Δ*S*_3_
O_M_	0.91 ± 0.15	−8.24 ± 0.10	−40.1 ± 1.7	−31.9 ± 1.8	3.73 ± 0.72	−7.40 ± 0.12	−30.2 ± 5.5	−22.8 ± 5.6	—	—	—	—
O_LM_	0.48 ± 0.09	−8.62 ± 0.11	−68.0 ± 1.5†	−51.1 ± 1.8‡	1.03 ± 0.21	−8.17 ± 0.15	nd†	nd‡	53 ± 53	−5.84 ± 0.60	nd§	nd§
O_MR_	0.22 ± 0.11	−9.09 ± 0.25	−60.8 ± 2.3†	−43.4 ± 2.6‡	0.89 ± 0.48	−8.25 ± 0.32	nd†	nd‡	1.52 ± 0.22	−7.94 ± 0.20	−86 ± 28	−78 ± 28

† Refers to the sum Δ*H*_1_ + Δ*H*_2_. In the case of O_LM_ and O_RM_ titrations it is not possible to reliably determine Δ*H*_1_ and Δ*H*_2_ separately as they are strongly correlated. The same holds for the respective entropic contributions. However, the total sum (Δ*H*_1_ + Δ*H*_2_) can reliably be obtained from fitting. Note that the obtained sums are comparable to that for the titrations with O_M_ (Δ*H*_1_ + Δ*H*_2_ = −70.3 kcal mol^−1^), which correspond to the same binding events to the central inverted repeat via the first and second YdaT.

‡ Refers to the sum *T*Δ*S*_1_ + *T*Δ*S*_2_.

§ The affinity for the second operator binding site is too weak (*K*_d3_ = 53 µ*M*) to reliably determine Δ*H*_3_.

## References

[bb1] Afonine, P. V., Grosse-Kunstleve, R. W., Echols, N., Headd, J. J., Moriarty, N. W., Mustyakimov, M., Terwilliger, T. C., Urzhumtsev, A., Zwart, P. H. & Adams, P. D. (2012). *Acta Cryst.* D**68**, 352–367. 10.1107/S0907444912001308 PMC3322595 22505256

[bb2] Agarwal, S. & Cho, T. Y. (2018). *Nucleic Acids Res.* **46**, 929–941. 10.1093/nar/gkx1183 PMC5778499 29186613

[bb3] Altschul, S. F., Madden, T. L., Schäffer, A. A., Zhang, J., Zhang, Z., Miller, W. & Lipman, D. J. (1997). *Nucleic Acids Res.* **25**, 3389–3402. 10.1093/nar/25.17.3389 PMC146917 9254694

[bb4] Brüssow, H. & Kutter, E. (2004). *Bacteriophages: Biology and Applications*, edited by E. Kutter & A. Sulakvelidze, pp. 91–128. Boca Raton: CRC Press.

[bb5] Campbell, A. (1994). *Annu. Rev. Microbiol.* **48**, 193–222. 10.1146/annurev.mi.48.100194.001205 7826005

[bb6] Casjens, S. (2003). *Mol. Microbiol.* **49**, 277–300. 10.1046/j.1365-2958.2003.03580.x 12886937

[bb7] Christensen-Dalsgaard, M., Jørgensen, M. G. & Gerdes, K. (2010). *Mol. Microbiol.* **75**, 333–348. 10.1111/j.1365-2958.2009.06969.x PMC2814082 19943910

[bb8] Chung, S. & Echols, H. (1977). *Virology*, **79**, 312–319. 10.1016/0042-6822(77)90358-0 867825

[bb9] Court, D. L., Oppenheim, A. B. & Adhya, S. L. (2007). *J. Bacteriol.* **189**, 298–304. 10.1128/JB.01215-06 PMC1797383 17085553

[bb10] Datta, A. B., Panjikar, S., Weiss, M. S., Chakrabarti, P. & Parrack, P. (2005). *Proc. Natl Acad. Sci. USA*, **102**, 11242–11247. 10.1073/pnas.0504535102 PMC1183575 16061804

[bb11] David, G. & Pérez, J. (2009). *J. Appl. Cryst.* **42**, 892–900.

[bb12] Emsley, P., Lohkamp, B., Scott, W. G. & Cowtan, K. (2010). *Acta Cryst.* D**66**, 486–501. 10.1107/S0907444910007493 PMC2852313 20383002

[bb13] Gasteiger, E., Hoogland, C., Gattiker, A., Duvaud, S., Wilkins, M. R., Appel, R. D. & Bairoch, A. (2005). *The Proteomics Protocols Handbook*, edited by J. M. Walker, pp. 571–607. Totowa: Humana Press.

[bb14] Goeders, N. & Van Melderen, L. (2014). *Toxins*, **6**, 304–324. 10.3390/toxins6010304 PMC3920263 24434905

[bb15] Hallez, R., Geeraerts, D., Sterckx, Y., Mine, N., Loris, R. & Van Melderen, L. (2010). *Mol. Microbiol.* **76**, 719–732. 10.1111/j.1365-2958.2010.07129.x 20345661

[bb16] Ho, Y. S. & Rosenberg, M. (1985). *J. Biol. Chem.* **260**, 11838–11844. 2931430

[bb17] Jacobson, E. M., Li, P., Leon-del-Rio, A., Rosenfeld, M. G. & Aggarwal, A. K. (1997). *Genes Dev.* **11**, 198–212. 10.1101/gad.11.2.198 9009203

[bb18] Jain, D., Kim, Y., Maxwell, K. L., Beasley, S., Zhang, R., Gussin, G. N., Edwards, A. M. & Darst, S. A. (2005). *Mol. Cell*, **19**, 259–269. 10.1016/j.molcel.2005.06.006 16039594

[bb19] Jauch, R., Choo, S. H., Ng, C. K. L. & Kolatkar, P. R. (2011). *Proteins*, **79**, 674–677. 10.1002/prot.22916 21117060

[bb20] Jobling, M. G. (2018). *mSphere*, **3**, e00163-18.

[bb21] Johnson, A. D., Poteete, A. R., Lauer, G., Sauer, R. T., Ackers, G. K. & Ptashne, M. (1981). *Nature*, **294**, 217–223. 10.1038/294217a0 6457992

[bb22] Jurėnas, D., Fraikin, N., Goormaghtigh, F., De Bruyn, P., Vandervelde, A., Zedek, S., Jové, T., Charlier, D., Loris, R. & Van Melderen, L. (2021). *mBio*, **12**, e02947-21. 10.1128/mBio.02947-21 PMC8630535 34844426

[bb23] Kabsch, W. (2010). *Acta Cryst.* D**66**, 125–132. 10.1107/S0907444909047337 PMC2815665 20124692

[bb24] Kaiser, K. & Murray, N. E. (1979). *Mol. Gen. Genet.* **175**, 159–174. 10.1007/BF00425532 390313

[bb25] Kobiler, O., Koby, S., Teff, D., Court, D. & Oppenheim, A. B. (2002). *Proc. Natl Acad. Sci. USA*, **99**, 14964–14969. 10.1073/pnas.222172499 PMC137528 12397182

[bb26] Krishnamurthi, R., Ghosh, S., Khedkar, S. & Seshasayee, A. S. N. (2017). *mSphere*, **2**, e00392-17. 10.1128/mSphere.00392-17 PMC5700373 29205228

[bb27] Maxam, A. & Gilbert, W. (1980). *Methods Enzymol.* **65**, 499–560. 10.1016/s0076-6879(80)65059-9 6246368

[bb28] McCoy, A. J., Grosse-Kunstleve, R. W., Adams, P. D., Winn, M. D., Storoni, L. C. & Read, R. J. (2007). *J. Appl. Cryst.* **40**, 658–674. 10.1107/S0021889807021206 PMC2483472 19461840

[bb29] Michael, A. K., Grand, R. S., Isbel, L., Cavadini, S., Kozicka, Z., Kempf, G., Bunker, R. D., Schenk, A. D., Graff-Meyer, A., Pathare, G. R., Weiss, J., Matsumoto, S., Burger, L., Schübeler, D. & Thomä, N. H. (2020). *Science*, **368**, 1460–1465. 10.1126/science.abb0074 32327602

[bb30] Panjkovich, A. & Svergun, D. I. (2018). *Bioinformatics*, **34**, 1944–1946. 10.1093/bioinformatics/btx846 PMC5972624 29300836

[bb31] Pereira, J. H. & Kim, S.-H. (2009). *J. Struct. Biol.* **167**, 159–165. 10.1016/j.jsb.2009.05.003 19450691

[bb32] Perna, N. T., Plunkett, G., Burland, V., Mau, B., Glasner, J. D., Rose, D. J., Mayhew, G. F., Evans, P. S., Gregor, J., Kirkpatrick, H. A., Pósfai, G., Hackett, J., Klink, S., Boutin, A., Shao, Y., Miller, L., Grotbeck, E. J., Davis, N. W., Lim, A., Dimalanta, E. T., Potamousis, K. D., Apodaca, J., Anantharaman, T. S., Lin, J., Yen, G., Schwartz, D. C., Welch, R. A. & Blattner, F. R. (2001). *Nature*, **409**, 529–533. 10.1038/35054089 11206551

[bb33] Phillips, K. & Luisi, B. (2000). *J. Mol. Biol.* **302**, 1023–1039. 10.1006/jmbi.2000.4107 11183772

[bb35] Ptashne, M., Jeffrey, A., Johnson, A. D., Maurer, R., Meyer, B. J., Pabo, C. O., Roberts, T. M. & Sauer, R. T. (1980). *Cell*, **19**, 1–11. 10.1016/0092-8674(80)90383-9 6444544

[bb36] Reményi, A., Tomilin, A., Pohl, E., Lins, K., Philippsen, A., Reinbold, R., Schöler, H. R. & Wilmanns, M. (2001). *Mol. Cell*, **8**, 569–580. 10.1016/s1097-2765(01)00336-7 11583619

[bb37] Russo, S., Schweitzer, J. E., Polen, T., Bott, M. & Pohl, E. (2009). *J. Biol. Chem.* **284**, 5208–5216. 10.1074/jbc.M806591200 19019826

[bb38] Scheuermann, T. H. & Brautigam, C. A. (2015). *Methods*, **76**, 87–98. 10.1016/j.ymeth.2014.11.024 PMC4380771 25524420

[bb39] Schwieters, C. D., Bermejo, G. A. & Clore, G. M. (2018). *Protein Sci.* **27**, 26–40. 10.1002/pro.3248 PMC5734396 28766807

[bb40] Schwieters, C. D. & Clore, G. M. (2014). *Prog. Nucl. Magn. Reson. Spectrosc.* **80**, 1–11. 10.1016/j.pnmrs.2014.03.001 PMC4057650 24924264

[bb41] Schwieters, C. D., Kuszewski, J. J., Tjandra, N. & Clore, G. M. (2003). *J. Magn. Reson.* **160**, 65–73. 10.1016/s1090-7807(02)00014-9 12565051

[bb42] Sevin, E. W. & Barloy-Hubler, F. (2007). *Genome Biol.* **8**, R155. 10.1186/gb-2007-8-8-r155 PMC2374986 17678530

[bb43] Tataurov, A. V., You, Y. & Owczarzy, R. (2008). *Biophys. Chem.* **133**, 66–70. 10.1016/j.bpc.2007.12.004 18201813

[bb44] Tickle, I. J., Flensburg, C., Keller, P., Paciorek, W., Sharff, A., Vonrhein, C. & Bricogne, G. (2018). *STARANISO*. Global Phasing Ltd, Cambridge, United Kingdom.

[bb45] Vandervelde, A., Drobnak, I., Hadži, S., Sterckx, Y. G., Welte, T., De Greve, H., Charlier, D., Efremov, R., Loris, R. & Lah, J. (2017). *Nucleic Acids Res.* **45**, 2937–2950. 10.1093/nar/gkx108 PMC5389731 28334797

[bb46] Wegrzyn, G. & Wegrzyn, A. (2005). *Prog. Nucleic Acid Res. Mol. Biol.* **79**, 1–48. 10.1016/S0079-6603(04)79001-7 16096026

[bb47] Wen, Y., Behiels, E., Felix, J., Elegheert, J., Vergauwen, B., Devreese, B. & Savvides, B. (2014). *Nucleic Acids Res.* **42**, 10134–10147. 10.1093/nar/gku665 PMC4150777 25056321

