# Supplementary material for: Structural basis of DNA binding by YdaT, a functional equivalent of the CII repressor in the cryptic prophage CP-933P from Escherichia coli O157:H7
Source: Acta Crystallogr D Struct Biol. 2023 Feb 27;79(Pt 3):245–58. doi: 10.1107/S2059798323001249 (PMC9986795; doi:10.1107/S2059798323001249)
Supplement: Supplementary file 1 [file d-79-00245-sup1.pdf]

STRUCTURAL
BIOLOGY

Volume 79 (2023)

Supporting information for article:

Structural basis of DNA binding by YdaT, a functional equivalent of the CII repressor in the cryptic prophage CP-933P from *Escherichia coli* O157:H7

Maruša Prolič-Kalinšek, Alexander N. Volkov, San Hadži, Jeroen Van Dyck, Indra Bervoets, Daniel Charlier and Remy Loris

Table S1 Primers used for cloning, EMSA and to generate the fragment used for footprinting

primer	sequence 5' -> 3'
1	AGCCACAGTTAAGAATATTACCGTGAGATCGTCGA
2	TAATATTCTTAACTGTGGCTGCGCATACGGGC
3	GGGATCGGAACGTGAAGGATGTCGATGATCGTGTTGCGTCAGCGGTTGTTTTGTATGAC
4	ACGCAACACGATCATCGACATCCTTCACGTTCCGATCCCTCCGTTCGACGATCTCACGG
5	TTCGCAGCCGGTACTTTTTC
6	GGAGCTACGACCCCATGATT
7	ATTCCCAGCTCGAAATACGCT

Table S2 Crystallographic data collection and dataprocessing

Values for the outer shell are given in parentheses.

(a) Data collection and scaling using XDS	
Diffraction source	Soleil Proxima 1,
Wavelength (Å)	0.976
Temperature (K)	100
Detector	EIGER X 9M
Crystal-detector distance (mm)	331.98
Rotation range per image (°)	0.1
Total rotation range (°)	240
Space group	P1
a, *b*, *c* (Å)	45.57, 64.26, 63.91
α, β, γ (°)	66.83, 89.36, 77.67
Mosaicity (°)	0.193
Resolution range (Å)	44.37 - 2.40 (2.52-2.40)
(b) Anisotropic scaling using Staraniso	
Ellipsoidal resolution (Å)	
along 0.880 **a*** + 0.221 **b*** - 0.420 **c***	2.23
along 0.072 **a*** + 0.854 **b*** + 0.515 **c***	2.40
along 0.292 **a*** - 0.214 **b*** + 0.932 **c***	2.90
Wilson B-factor (Å^2)	
along 0.856 **a*** + 0.296 **b*** - 0.424 **c***	34.79
along 0.012 **a*** + 0.887 **b*** + 0.462 **c***	54.93
along 0.323 **a*** - 0.043 **b*** + 0.946 **c***	103.30
Completeness (spherical)	77.4 (32.5)
Completeness (ellipsoidal)	90.9 (72.8)
R_{merge}	0.082 (0.386)
R_{meas}	0.106 (0.552)
CC(1/2)	0.990 (0.797)
Total No. of reflections	45947 (2046)
No. of unique reflections	19698 (993)
⟨ *I*/σ(*I*)⟩	6.0 (1.4)

Table S3 Crystallographic structure solution and refinement

Resolution range (Å)	44.40 - 2.40 (2.52-2.40)
σ cutoff	none
No. of reflections, working set	18719
No. of reflections, test set	979
Final R_{cryst}	0.2112 (0.2757)
Final R_{free}	0.2658 (0.2783)
No. of non-H atoms	
Protein	3757
Other	154
R.m.s. deviations	
Bond lengths (Å)	0.0088
Bond angles (°)	0.959
Chirality	0.052
Clashscore	5.83
Planar groups	0.055
Rotamer outliers (%)	3.17
Overall Molprobity score	1.82
Average B factor (Å^2)	56.0
Protein	84.57
Ramachandran plot	
Most favoured (%)	97.4
Additionally allowed	2.0
Disallowed (%)	0.6
PDB deposition	8bt1

Statistics for the highest-resolution shell are shown in parentheses.

Table S4 SAXS experimental details

	YdaT	YdaT$^{1\text{-}96}$	YdaT-O_M
(*a*) Sample details			
Loading volume	45 µl	45 µl	45 µl
Injected concentration	10 mg ml^{-1}	10 mg ml^{-1}	~8 mg ml^{-1} *
Flow rate	0.3 ml min^{-1}	0.3 ml min^{-1}	0.16 ml min^{-1}
Solvent composition	20 mM Tris-HCl, 200 mM NaCl, pH 8	20 mM Tris-HCl, 200 mM NaCl, pH 8	20 mM Tris-HCl, 200 mM NaCl, pH 8
(*b*) SAS data collection parameters			
Source	SWING beamline, SOLEIL	SWING beamline, SOLEIL	BM29 beamline, ESRF
Sample configuration	In-line HPLC, quartz capillary cell	In-line HPLC, quartz capillary cell	In-line HPLC, quartz capillary cell
Wavelength (Å)	1.03	1.03	0.99
q-measurement range (Å^{-1})	0.0037 – 0.52	0.0037 – 0.51	0.0044 – 0.52
Exposure time, number of exposures	0.990 s frames	0.990 s frames	2.0 s frames
Sample configuration	In-line HPLC, quartz capillary cell	In-line HPLC, quartz capillary cell	In-line HPLC, quartz capillary cell
Sample temperature (ºC)	15	15	15
(*c*) Software			
Data reduction	Foxtrot 3.5.10-3979	Foxtrot 3.5.10-3979	DAHU, CHROMIXS
Basic analyses: Guinier, *P*(*r*), Porod volume V_P)	Primus from the ATSAS 3.03 package	Primus from the ATSAS 3.03 package	Primus from the ATSAS 3.03 package
Atomic structure modelling	Xplor-NIH v 2.49	Xplor-NIH v 2.49	Xplor-NIH v 2.49
(*d*) Structural parameters			
Guinier analysis:			
I(0) (cm^{-1})	0.058 +/- 0.00004	0.021 +/- 0.00005	5.78 +/- 0.016
R_g (Å)	34.79 +/- 0.03	24.06 +/- 0.09	41.88 +/- 0.17
q-range (Å^{-1})	0.017 - 0.037	0.010 - 0.074	0.011 – 0.031
Quality of fit estimation (from AUTORG)	0.875	0.928	0.745
P(*r*) analysis:			
I(0) (cm^{-1})	0.06 +/- 0.00003	0.02 +/- 0.00004	5.77 +/- 0.013

R_g (Å)	34.94 +/- 0.03	24.75 +/-0.08	42.13 +/- 0.1
d_{max} (Å)	119.9	82.19	128.13
q-range (Å^{-1})	0.017 - 0.252	0.010 - 0.461	0.011 - 0.191
χ^2 (total estimate from *GNOM*)	0.8721	0.8315	0.9065
Volume (V_P, Å^3)	129558	28973	193022
(*e*) Atomistic modelling			
Method	Simulations in Xplor-NIH based on crystallographic coordinates of the tetramer YdaT	Simulations in Xplor-NIH based on crystallographic coordinates of the N-terminal YdaT domain	Simulations in Xplor-NIH based on crystallographic coordinates docked to DNA model
q-range for fitting (Å^{-1})	0.00620 - 0.24990	0.00660 - 0.24990	0.01110 - 0.22040
χ^2 value/range	1.684	0.99	1.418
(*f*) Deposition			
Deposition IDs	SASDQB9	SASDQD9	SASDQE9

* Approximate value - see Material and Methods for details regarding sample preparation

Table S5 **Operator fragments used for ITC, native MS and SAXS**. The complete and incomplete inverted repeats are underlined.

oligonucleotide	sequence 5' -> 3'
O_M	TCATGCTTGATTTTCATGAATCAACTCCAT
O_L	CCGGATGCGCCCAGACATTCATCGCC
O_R	GATAAGCATGCACTACAATCACCTTC
O_{LM}	GGATGCGCCCAGACATTCATCGCCATGCGGATGTGTTCATGCTTGATTTTCATGAATCAACT
O_{MR}	GCTTGATTTTCATGAATCAACTCCATCAGATAAGCATGCACTACAATCACCT

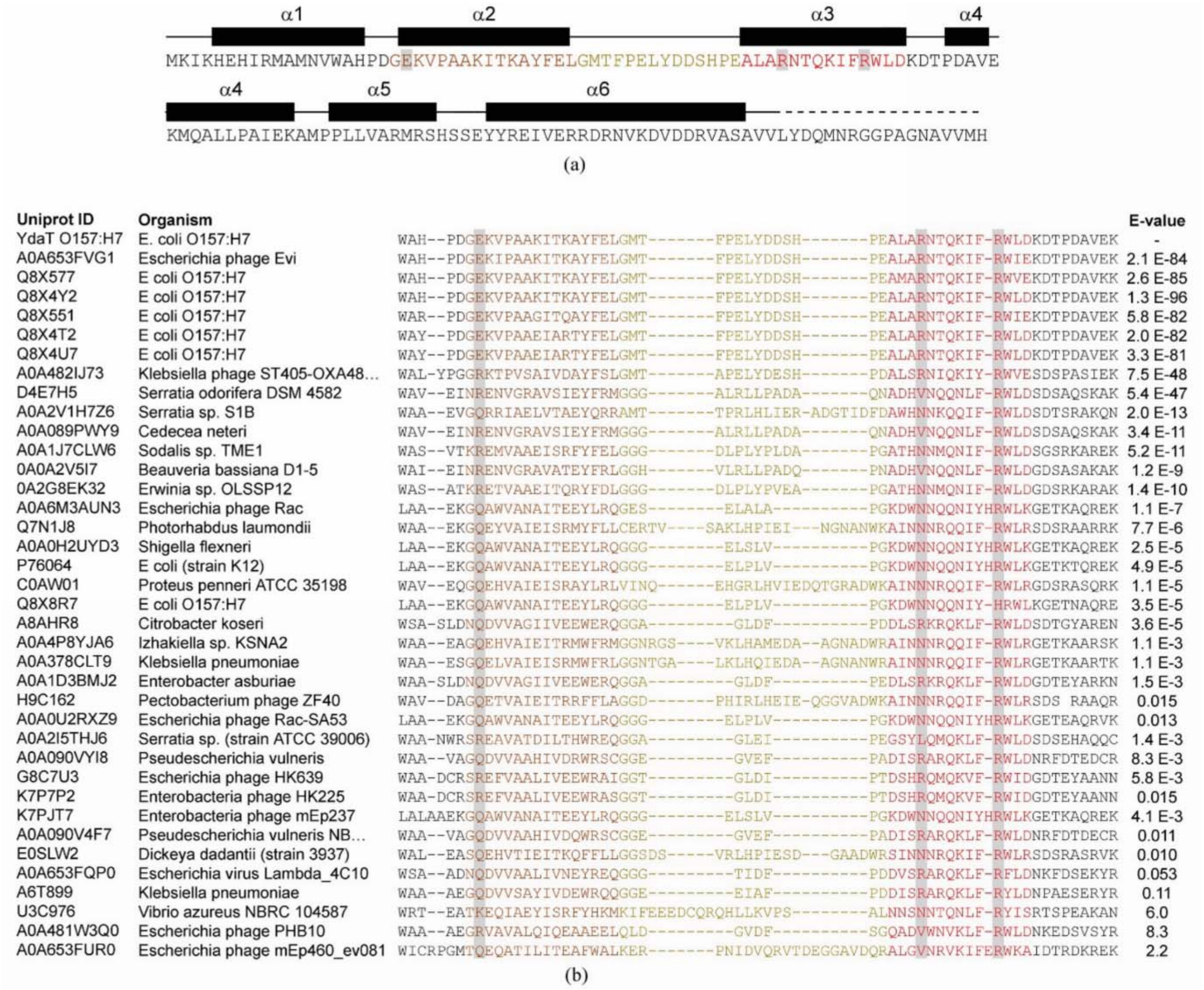

Figure S1 YdaT sequence. **a.** Comparison of the immunity region of bacteriophage λ and prophage CP-933P. **b.** Amino acid sequence of YdaT with the secondary structure elements indicated. The HTH motif is coloured with helix α2 in orange, the recognition helix α3 in red and the connecting loop in dark yellow. Residues Glu21, Arg53 and Arg60 are highlighted on a grey background **c.** Sequence alignment of the HTH motif of 38 selected homologs that were picked up using a BLAST search. Picked-up sequences that were (likely) incomplete or that according to Alphafold2 predictions deviate from the POU domain structure were removed. To obtain the best possible alignment of the α2-α3 loop region, Alphafold2 predictions were used as a guide. Colouring is as in panel a.

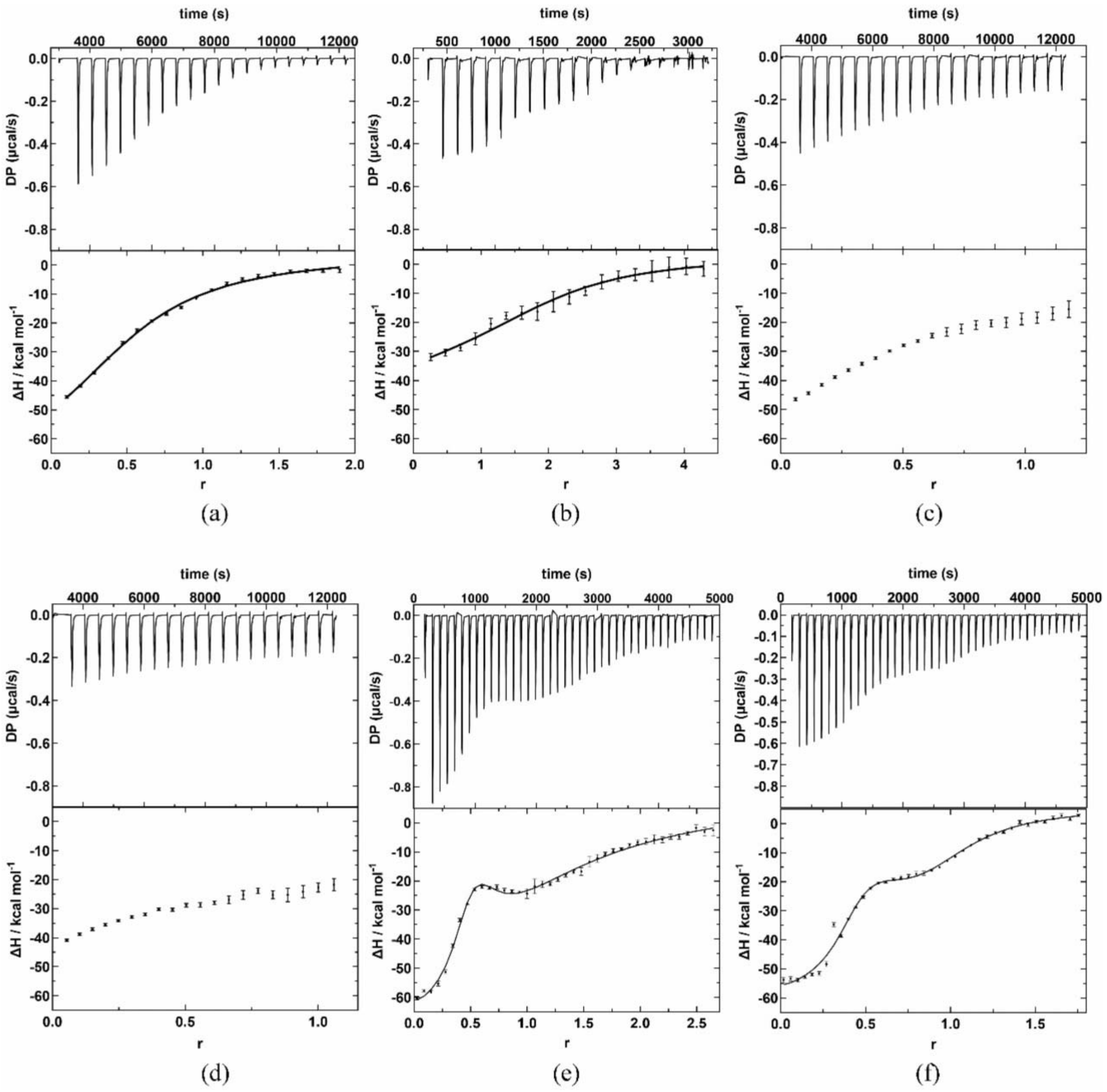

Figure S2 ITC analysis of DNA binding for YdaT at 25 °C. Upper panels show thermograms with subtracted baseline and lower panels integrated heats of injections per mole of injected protein or DNA. Black curves represent fitted model function. All molar ratios were calculated for YdaT as a tetramer. **a.** YdaT titrated into O_M solution. **b.** Reverse titration of O_M into YdaT. A model function for two independent non-equivalent binding sites was fitted globally to both titrations of YdaT and O_M yielding a single set of thermodynamic parameters. **c.-d.** Titrations of YdaT into O_L and O_R. **e.-f.** YdaT titrated into O_{LM} and $O_{MR.}$

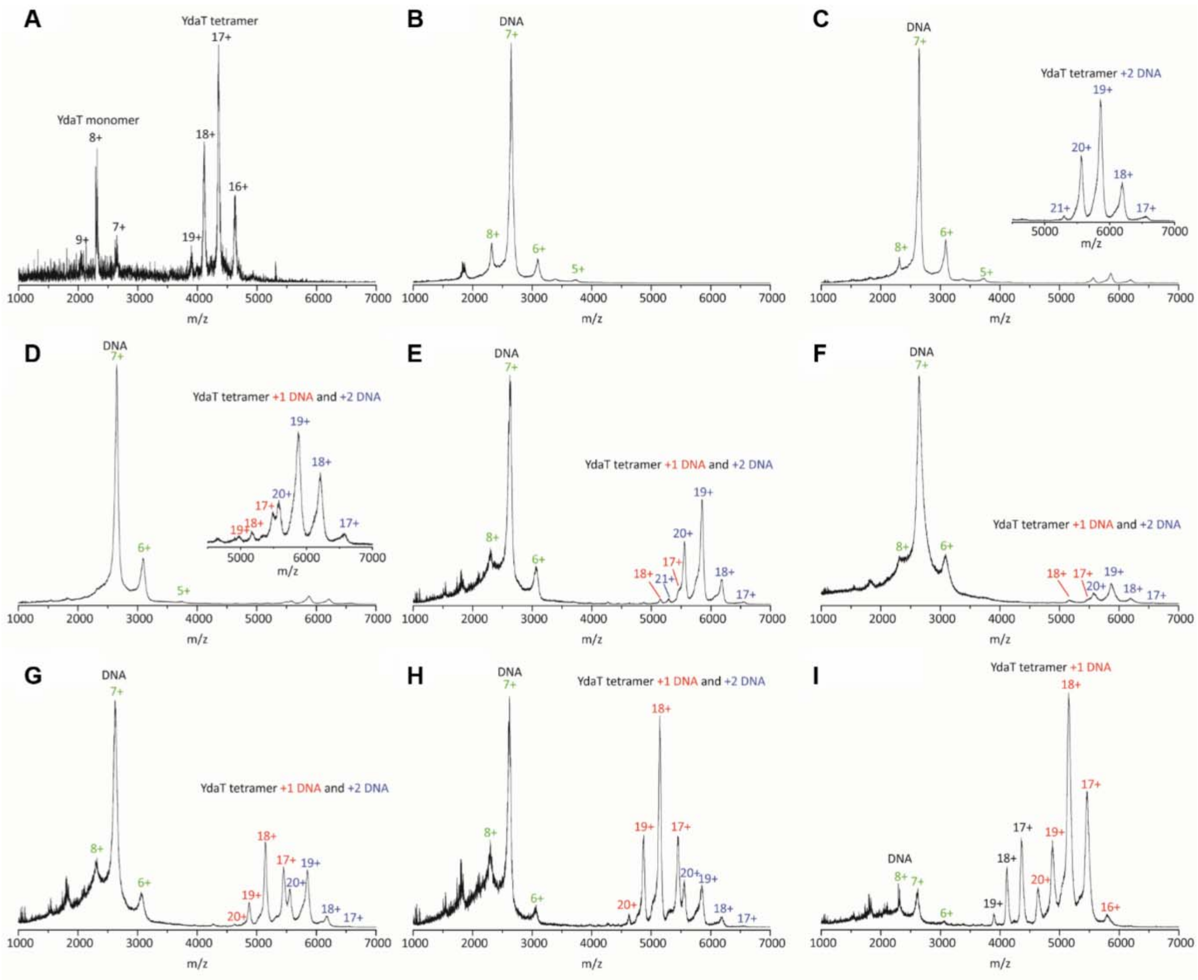

Figure S3 Native mass spectrometry of YdaT titrated with O_M**.** All concentrations are calculated for YdaT tetramer. **a.** Native MS spectrum of a free YdaT at a concentration of 2.5 µM (tetramer equivalents. **b.** Native MS spectrum for a free O_M duplex at a concentration of 5 µM. **c.-i.** Titration of O_M into 2.5 µM YdaT. O_M concentrations are in following order: 15 µM, 10 µM, 5 µM, 3.33 µM, 2.5 µM, 2 µM and 1.67 µM. When YdaT is oversaturated with O_M (c) only a species corresponding to $YdaT_4$-2xO_M is observed next to an excess of free O_M. With increasing protein to DNA ratio (d-h) a mixture of $YdaT_4$-2xO_M and $YdaT_4$-O_M is observed with the latter increasing. At a 1.5x excess of protein (i) only a species $YdaT_4$-O_M is observed in addition to free protein.

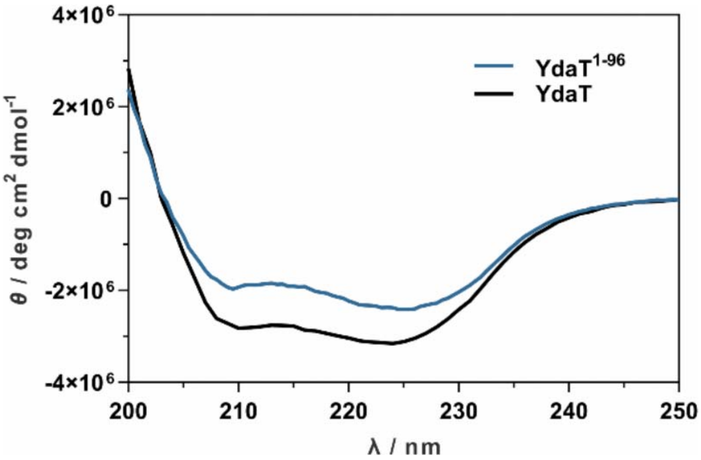

Figure S4 CD spectroscopy. CD spectra are shown for wild-type YdaT (black) and Yda^{T1-96} (blue), and were normalized in terms of molar equivalents of monomers All proteins show clear evidence for a folded structure dominated by α-helix (two minima at approximately 222 and 208 nm). The somewhat smaller signal for YdaT$^{1-96}$ reflects the absence of the C-terminal helix α6.

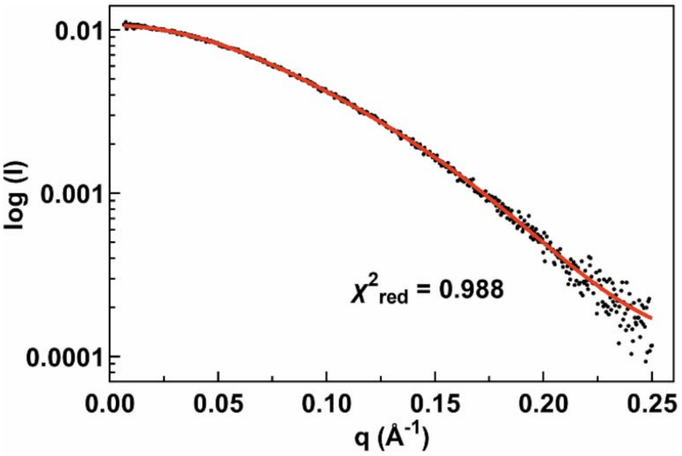

(a)

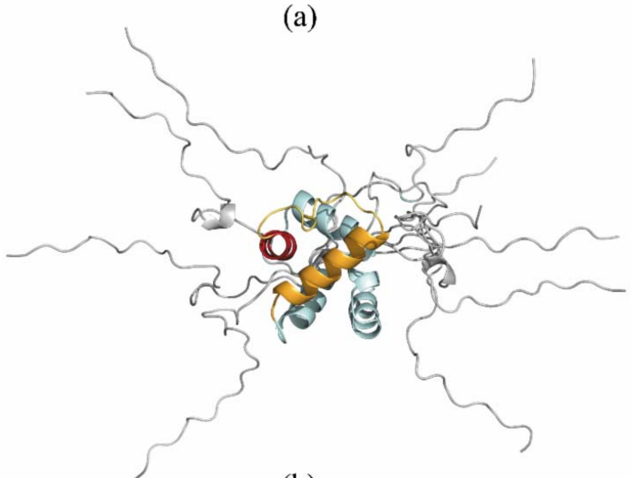

(b)

Figure S5 SAXS analysis of the YdaT mutants. **a.** Experimental SAXS data of YdaT$^{1-96}$ (black) with the calculated curve for the best ensemble of the protein (10 models, χ^2 0.983) superimposed in red. **b.** Superimposed ensemble of YdaT$^{1-96}$ models showing multiple orientations of the N-terminal His-tag and a rigid POU domain.
